# How Much Is It Weighing on You? Development and Validation of the Secrecy Burden Scale

**DOI:** 10.1177/01461672231172387

**Published:** 2023-05-24

**Authors:** Alisa Bedrov, Shelly L. Gable

**Affiliations:** 1 University of California, Santa Barbara, USA

**Keywords:** secrecy burden, measure, factor analysis, social relationships, well-being

## Abstract

Keeping a secret is often considered burdensome, with numerous consequences for well-being. However, there is no standardized measure of secrecy burden, and most studies focus on individual/cognitive burden without considering social/relational aspects. This research aimed to develop and validate a secrecy burden measure tapping both intrapersonal and interpersonal components. Study 1 used exploratory factor analysis to reveal a four-factor model of secrecy burden: Daily Personal Impact, Relationship Impact, Pull to Reveal, and Anticipated Consequences. Study 2 used confirmatory factor analysis to replicate this factor structure and found that each factor was uniquely associated with different emotional and well-being outcomes. Study 3 employed a longitudinal design and found that higher scores on each factor predicted lower authenticity and higher depression and anxiety 2 to 3 weeks later. Altogether, this research is the first step in standardizing a secrecy burden measure and applying it to real-world secrets and well-being outcomes.

Most everyone is familiar with the discomfort of keeping a secret. If the topic comes up in conversation, one must avoid revealing the information, and even outside of face-to-face interactions, there is a continuous predicament of deciding when and how to reveal the information (if ever). Indeed, the colloquial phrase “to get something off my chest” speaks to the often-burdensome nature of secrecy. Some secrets are benign and harmless, whereas other secrets are significantly heavier and more consequential. Just as there is variability in the types of secrets that people keep, there is also variation in the extent that a given secret may be burdensome and consequential for personal and relational well-being. The purpose of this research is to (a) use exploratory and confirmatory factor analysis to develop a scale that assesses the burden of keeping a secret and (b) apply the scale to understand how different facets of secrecy burden relate to personal and relational well-being.

## Prior Measures of Secrecy Burden

Most research examining secrets and subsequent well-being refers to secrecy as a *burden*. Whether it be one’s own secret or someone else’s, the literature assumes that withholding information is burdensome due to self-regulatory demands and preoccupying thoughts (e.g., Critcher & Ferguson, 2013; Lane & Wegner, 1995), and most research has focused on how secrecy burden and preoccupation correspond to negative effects on the secret-keeper’s well-being (e.g., Slepian et al., 2017; Zhang & Dailey, 2018). However, almost no attention has been allocated to establishing a standardized measure of secrecy burden. Research employing self-report measures has assessed burden using the cognitive preoccupation subscale of the Tilburg Secrecy Scale-25 (TSS-25; Zhang & Dailey, 2018) or by averaging one or two statements like “I wish I never learned this secret” and “I feel burdened by this secret” (Slepian & Greenaway, 2018). Other research has used single-item measures assessing the extent of effort and difficulty involved in keeping the secret (Bedrov & Leary, 2021). Burden has also been inferred from theoretically-related constructs, such as judgments of distance and hill steepness (Slepian et al., 2012, 2015, 2016). Critcher and Ferguson (2013) similarly inferred secrecy burden from depletion on self-regulatory tasks regarding intelligence, politeness, and physical strength. All of these conceptualizations presumably measure the same construct of “secrecy burden,” yet such variability in measurement increases the difficulty of aggregating related findings and reaching a consensus as to how secrecy burden affects the individual.

Nevertheless, secrecy burden is important to study given evidence of its negative effects on personal well-being and relationships. Setting measurement differences aside, high secrecy burden has been associated with lower well-being and decreased relationship satisfaction (Bedrov & Leary, 2021; Slepian et al., 2017), as well as increased fatigue, negative affect, and social isolation (Slepian et al., 2019; Zhang & Dailey, 2018). Keeping a secret clearly has important consequences for well-being, yet there remains a dire need for a standardized measure of secrecy burden so as to better understand and address these consequences.

Furthermore, prior measures of secrecy burden have almost exclusively focused on *individual* aspects of concealment, that is, the secret-keeper’s cognitive and emotional capacity to cope with the demands of secrecy. Yet, secrets are inherently *social* in that the burden stems from not only the secret-keeper’s process of concealment but also how keeping the secret affects their relationship with the person from whom the secret is kept (i.e., the target). People often experience distress and relationship dissatisfaction when lying to or withholding information from close rather than distant others (DePaulo & Kashy, 1998; Uysal et al., 2012), suggesting that secrecy burden depends on existing relationship closeness. Slepian and Greenaway (2018) also found that keeping another person’s secret was perceived as more burdensome when there was greater overlap between social networks, as high commonality in friendship circles would likely lead to more instances of concealment. Thus, secrecy burden and its subsequent effects on well-being cannot be fully understood without also considering the social demands and consequences of concealment.

## Conceptualizing Secrecy Burden as Multi-Faceted

Given that secrecy burden has both individual and social aspects, we propose that secrecy burden is a multi-faceted construct and should be measured accordingly. Consistent with prior research, the first facet should encompass the individual cognitions and mental resources involved in the intent to withhold information from others. Such burden manifests as frequent mind-wandering and rumination (e.g., Slepian et al., 2017), perceived stress and difficulty of keeping the secret (e.g., Bedrov & Leary, 2021), and any personal adjustments made to everyday life that facilitate keeping the secret. Second, people often worry about the potential consequences of revealing their secret, and perceiving greater potential costs upon revealing is associated with more preoccupying thoughts (Davis & Brazeau, 2021), thus creating a more future-oriented source of burden. These first two facets (investing mental resources and worrying about potential consequences) reflect some of the *individual* aspects of burden.

However, secrets are always kept from at least one person, and the secret-keeper’s relationship with that target can be another source of burden. For instance, keeping a secret can lead to greater inauthenticity or relationship dissatisfaction (e.g., Newheiser & Barreto, 2014; Uysal et al., 2012), which may make the secret-keeper distressed over how the secret is impacting their relationship. Furthermore, people generally value and expect openness and self-disclosure from others (e.g., Goodwin et al., 2014; Hall, 2012; Sprecher et al., 2013), with secrecy often being a violation of those expectations. In turn, the secret-keeper may feel guilty or experience burden from juggling the social pressures toward openness and their personal desire for non-disclosure. Thus, these next two facets (the secret’s relational implications and social pressures to reveal it) capture the *social* aspects of secrecy burden.

In addition to being multi-faceted, secrecy burden may also vary widely across individuals, secrets, or the different relationships in which the same secret is kept. By having a standardized measure of secrecy burden, we can better address questions like:

- What individual differences might make someone more likely to experience higher secrecy burden when keeping a secret?
- Do different types of secrets result in different levels of secrecy burden?
- To what extent does the level of secrecy burden vary depending on who the target of the secret is and what that relationship is like?

At the present moment, research has yet to have a reliable way of holistically assessing secrecy burden to allow for such questions to be investigated. A necessary first step is to understand and assess *how* people experience secrecy burden.

## The Current Research

Given that most research has focused on individual aspects of secrecy burden in isolation and has treated it as a unidimensional construct, this research aims to develop and validate a new measure that captures both individual and social aspects of secrecy burden. In Study 1, we use exploratory factor analysis (EFA) to examine whether emergent factors distinguish between individual and social dimensions of burden. We also examine the measure’s convergent and discriminatory validity relative to prior measures of secrecy burden. In Study 2, we use confirmatory factor analysis (CFA) in an independent sample to replicate the factor structure and examine correlations among the emergent factors and relevant well-being outcomes. In Study 3, to further examine predictive validity, we use the new measure to assess each subscale’s association with changes in personal and relational well-being while keeping a personal secret across two weeks. Our goal is to better define the construct of secrecy burden and bring the social aspects of secrecy to the forefront of theory and application.

## Study 1: Exploratory Factor Analysis

### Method

We report all measures and exclusions in these studies; all materials, data, and syntax are available at https://dataverse.harvard.edu/dataset.xhtml?persistentId=doi:10.7910/DVN/3OW2SS.

#### Participants

Participants were recruited via Prolific to complete a brief online study on how keeping a personal secret has impacted their lives. Sample size was based on recommendations of a minimum sample of 200 or a 10:1 participant-to-variable ratio for accurate EFA (Howard, 2016; Kyriazos, 2018; Widaman, 2012). We oversampled to account for participant attrition and maximize accuracy since factor analysis is highly dependent on large samples (Kyriazos, 2018). Participants were excluded for not currently keeping a negative personal secret (*n* = 11). The final sample (*N* = 299, age range = 18-70) was 49.16% female and predominantly White (59.20%), followed by Black/African American (18.06%), Hispanic (6.02%), other (4.35%), Asian/Pacific Islander (3.68%), and Middle Eastern (2.68%). Prolific has a wide global distribution, and participants’ current countries (in descending order of frequency) included the United Kingdom, South Africa, United States, Portugal, Poland, and Ireland, among others. Participants received U.S.$1.60 for completing the study.

#### Procedure

To be eligible for the study, participants had to currently be keeping a personal secret about themselves from at least one person. After reflecting on this secret, they completed the 18-item Secrecy Burden Scale, along with other questions about how long they had been keeping the secret and how important, serious, and personal the information was. Participants then completed measures of ruminative thought (Afifi & Caughlin, 2006; adopted from Scott & McIntosh, 1999), the cognitive preoccupation and apprehension about disclosure subscales of the TSS-25 (Maas et al., 2012; Van Vessem, 2010), state authenticity (Fleeson & Wilt, 2010), and the Positive and Negative Affect Scale (PANAS; Watson & Clark, 1994). Here, we focus on ruminative thought and the TSS, as both measures have been used in prior studies to assess secrecy burden (e.g., Afifi & Caughlin, 2006; Maas et al., 2012; Zhang & Dailey, 2018) and would allow us to test the new measure’s convergent and discriminant validity. Analyses of additional measures appear in the Supplemental material.

#### Measures

##### Secrecy Burden Scale

Items for this measure were generated based on previous assessments of secrecy burden (e.g., Bedrov & Leary, 2021; Slepian et al., 2017; Zhang & Dailey, 2018), the broader literature on secrecy and relevant theories, and responses from cognitive interviews on people’s experiences with keeping personal secrets. A series of pilot studies narrowed down the final measure to 18 items that captured both individual and social aspects of keeping a secret (see the Supplemental material). Briefly, we extracted common themes from cognitive interviews that reflected burdensome aspects of keeping a personal secret. We then created items that reflected those themes and captured both individual and social aspects of secrecy burden that should also affect well-being (e.g., mind-wandering frequency, anticipated consequences, relationship impact, social expectations for disclosure). Across two pilot studies (*N* = 871), we tested 25 items and used principal component analysis with varimax rotation to eliminate ill-fitting items (*n* = 7).

To begin, participants were asked to think of a personal secret about themselves that they were currently keeping from one or more people (even if some people already knew the information). They then responded to the items on 7-point Likert-type scales, and items were presented together based on their leading prompts. For example, the prompt “Over the past week . . .” was followed by items, such as “How often did you think about the secret?” (1 = *not at all*, 7 = *extremely often*). Another prompt, “When thinking about the people closest to you from whom you are keeping this secret . . .,” was followed by items, such as “To what extent do you feel guilty about keeping this secret from them?” (1 = *not at all*, 7 = *extremely*). See the Supplemental material for all prompts, items, and response scales. Five items were reverse coded: anticipated consequences of revealing, whether one’s life would become better/worse, feelings of authenticity, ease of interaction with people who do not know the secret, and whether one feels more distant or closer in those relationships. Table 1 shows the full scale with item means and standard deviations in the order presented to participants. Most items correlated with one another, with significant correlations ranging from *r* = –.13 (*p* < .01) to *r* = .68 (*p* < .001). The two items assessing anticipated consequences of revealing and how life would become better or worse were significantly correlated with the other two items assessing possible outcomes upon revealing but were largely uncorrelated with the rest of the items. We decided to retain these items for the EFA. The full table of all bivariate correlations among the 18 items can be found in the Supplemental material.

**Table 1.** Secrecy Burden Scale With Study 1 Means & Standard Deviations.

Item name	Full item	*M* (*SD*)
*Over the past week* . . .		
1. Ruminate	How often did you think about the secret?	4.36 (1.65)
2. Stress	To what extent was the secret a source of stress in your everyday life?	3.86 (1.74)
3. Effort	How much effort did you invest in keeping the secret?	4.54 (1.82)
4. Difficulty	How difficult was keeping the secret for you?	3.54 (1.85)
*Over the course of keeping this secret* . . .		
5. Distraction	How often have thoughts of this secret distracted you from daily activities?	3.41 (1.65)
6. Life adjust	To what extent have you had to make adjustments in your daily life to conceal this information?	3.23 (1.84)
7. Lying	How often have you had to lie to the people closest to you in order to keep this secret?	4.11 (1.92)
*When thinking about the people closest to you from whom you are keeping this secret. . .*		
8. Avoid social	How often have you had to avoid social situations because of this secret?	2.74 (1.83)
9. Distant^a^	To what extent has keeping this secret made you feel more distant or closer to them?^a^	4.77 (1.41)
10. Interact difficult^a^	To what extent has keeping this secret made your interactions with these people easier or more difficult?^a^	4.75 (1.28)
11. Authentic^a^	To what extent do you feel like your true authentic self when interacting with those people?^a^	3.58 (1.75)
12. Obligation	To what extent do you think they have a right or an obligation to know this secret?	3.01 (2.06)
13. Expectations	To what extent are you expected to share this type of information with them (i.e., the topic of your secret is something you would normally share with them)?	3.31 (1.98)
14. Guilt	To what extent do you feel guilty about keeping this secret from them?	3.70 (2.14)
*When thinking about potentially revealing this secret to the people closest to you who don’t know it . . .*		
15. Uncomf. Convo	To what extent would revealing the secret be an uncomfortable conversation to have?	5.64 (1.68)
16. Social reputation	Would revealing this secret negatively affect your social reputation/image?	4.07 (2.21)
17. Life better/ worse^a^	To what extent do you anticipate your life becoming better or worse because you no longer were keeping this a secret?^a^	4.30 (1.56)
18. Consequences^a^	Overall, what type of consequences do you anticipate from revealing this information?^a^	4.88 (1.50)

a Reverse-coded items. Item means were computed after reverse coding.

##### Ruminative Thought

Prior studies have used six items from Afifi and Caughlin’s (2006) adapted version of the Scott–McIntosh Rumination Inventory (SMRI; Scott & McIntosh, 1999) to assess secrecy burden. This version specifically focuses on ruminative tendencies related to distraction and emotionality for a currently-held secret; items include “I often get distracted from what I’m doing by thoughts of this secret” and “I become angry when I think about having to keep this secret from other people” (1 = *strongly disagree*, 7 = *strongly agree;* α = .535, *M* = 3.87, *SD* = 1.05). Although these items had poor reliability in our sample, we retained them to facilitate comparisons with prior work.

##### Tilburg Secrecy Scale

Participants responded to 10 items from the TSS-25, specifically the “cognitive preoccupation” and “apprehension about disclosure” subscales (1 = *strongly disagree*, 7 = *strongly agree*). An example from the cognitive preoccupation subscale is “I think about this secret often” (α = .891, *M* = 3.77, *SD* = 1.59). An example item from the apprehension about disclosure subscale is “If I tell my secret to my friends, they will like me less” (α = .849, *M* = 4.36, *SD* = 1.46). Higher scores on each subscale indicated higher secrecy-related apprehension and distress.

#### Analysis Plan

All exploratory factor analyses were conducted using the *MPlusAutomation* package in *R* (Hallquist & Wiley, 2018), which facilitated running models in Mplus 8.3 (Muthén & Muthén, 2017) through RStudio 4.1.2 (RStudio Team, 2021). Maximum likelihood estimation with robust standard errors (MLR) was used for the EFA to examine how close the generated factor solution was to the observed correlations between items while allowing for mild violations to the assumption of multivariate normality (Brown, 2015). Data were missing for only one participant’s response to one item, so that, we did not perform any list-wise deletions.

To allow correlations among emergent factors, we applied a geomin oblique rotation to all factor loadings. To determine the optimum number of factors for our model, we examined multiple fit criteria, including parallel analysis, Kaiser’s eigenvalues, comparative fit index (CFI), root mean-square error of approximation (RMSEA), and standardized root mean-square residual (SRMR). We also examined the chi-square test of model fit, with a non-significant chi-square indicating good fit. However, because chi-square is highly influenced by sample size (and our sample was relatively large), we expected the chi-square test to be significant and did not rely on it as a primary indicator of model fit (Fabrigar et al., 1999). For the parallel analysis, the optimum factor solution was identified as the number of factors with eigenvalues exceeding those generated from random data (i.e., the number of factors prior to the intersection of Cattell’s scree plot and the parallel analysis plot). Other criteria for good model fit were: Kaiser’s eigenvalues above 1.0, CFI = 0.90 to 0.95, RMSEA = 0.05 to 0.08, and SRMR = 0.05 to 0.08 (Brown, 2015; Fabrigar et al., 1999; Hu & Bentler, 1999). Items were considered to have a meaningful factor loading if the primary loading was above 0.30 and no cross-loadings above 0.30 (Brown, 2015).^1^ Items that failed to meet this criterion would be removed.

### Results

#### Data Screening

Prior to analyses, each item was screened for violations of normality using density plots and skewness and kurtosis statistics. Based on density plots, six items did not have a normal distribution (e.g., how revealing would affect social reputation had a bimodal distribution, obligation to reveal was positively skewed; see the Supplemental material for full details). However, although not all density plots showed evidence of normal distributions, all skewness and kurtosis values were below|2.0| and|7.0|, respectively, indicating no serious violations of normality (Curran et al., 1996). Maximum likelihood estimation with robust standard errors is robust to slight violations of normality, so that, we did not perform any data transformations on our items.

#### Exploratory Factor Analysis

**Figure 1.**
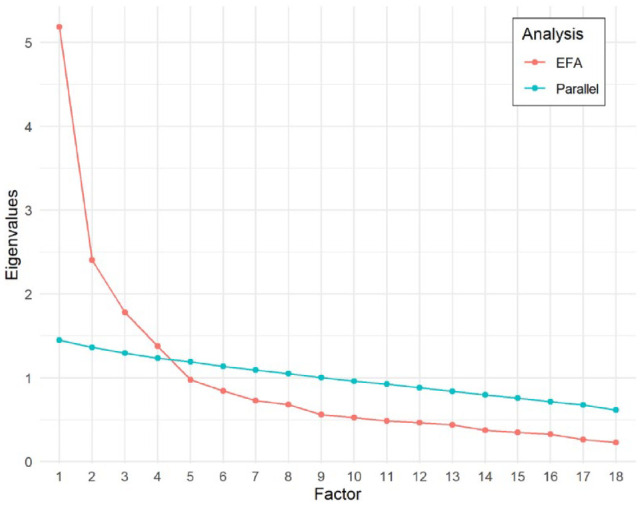
Results of the parallel analysis assessing model fit. *Note.* EFA = exploratory factor analysis.

Model solutions with factors ranging from one to five were investigated. All evaluations of model fit criteria supported a four-factor solution. Kaiser’s eigenvalues dropped below 1.0 at the fifth factor, and the parallel analysis plot intersected with Cattell’s scree plot between the fourth and fifth factors (see Figure 1).

Examination of model fit statistics provided further support for the four-factor model (see Table 2). The four-factor model had RMSEA = 0.066 [0.054, 0.078], CFI = 0.935, and SRMR = 0.035, all of which fell within the specified ranges for good model fit. The one-, two-, and three-factor models did not reach adequate fit, and although the five-factor model had better fit, this model was less parsimonious and not supported by the parallel analysis.^2^ Thus, we proceeded to examine the factor structure and loadings of the four-factor model.

**Table 2.** Summary of Model Fit Indices From EFA.

Model	Par	LL	χ^2^	*df*	*p* value	RMSEA [90% CI]	CFI	TLI	SRMR
One-factor	54	−10,099.930	916.847	135	< .001	0.139 [0.131, 0.148]	0.546	0.485	0.117
Two-factor	71	−9,926.469	611.445	118	< .001	0.118 [0.109, 0.128]	0.713	0.628	0.081
Three-factor	87	−9,789.922	351.056	102	< .001	0.090 [0.080, 0.101]	0.855	0.783	0.055
Four-factor	102	−9,707.825	198.836	87	< .001	0.066 [0.054, 0.078]	0.935	0.886	0.035
Five-factor	116	−9,669.298	136.637	73	< .001	0.054 [0.040, 0.068]	0.963	0.922	0.026

*Note.* EFA = exploratory factor analysis; LL = log likelihood; RMSEA = root mean-square error of approximation; CI = confidence interval; CFI = comparative fit index; TLI = Tucker–Lewis index; SRMR = standardized root mean-square residual.

Table 3 presents the geomin-rotated factor loadings for the four-factor model. All significant factor loadings were above our specified threshold of 0.30 (with all but one item also exceeding the more stringent cutoff of 0.40), and cross-loadings did not exceed 0.30. Thus, we retained all items in our final model. In addition, each factor had at least three or more indicators with strong primary loadings, suggesting no poorly defined factors (Brown, 2015).

**Table 3.** Summary of Geomin-Rotated Factor Loadings: 4-Factor EFA Model.

Names	*F*1	*F*2	*F*3	*F*4
Factor 1: Daily Personal Impact				
Ruminate	**0.686** *	−0.008	−0.049	−0.090
Stress	**0.730** *	−0.067	−0.040	−0.085
Effort	**0.672** *	0.075	0.005	0.131*
Difficulty	**0.622** *	0.061	0.156*	−0.005
Distraction	**0.698** *	−0.060	0.030	−0.092
Life adjust	**0.724** *	0.000	−0.071	0.054
Lying	**0.542** *	0.010	0.115	0.124*
Avoid social	**0.533** *	−0.141*	0.034	−0.079
Factor 2: Relationship Impact				
Distant	−0.008	**0.800** *	0.036	0.009
Interact difficult	0.015	**0.911** *	−0.019	0.001
Authentic	−0.150*	**0.444** *	−0.022	−0.122
Factor 3: Pressure to Reveal				
Obligation	−0.065	0.020	**0.908** *	−0.035
Expectations	0.101	0.027	**0.493** *	−0.140*
Guilt	0.269*	−0.068	**0.575** *	0.114*
Factor 4: Anticipated Consequences				
Uncomf. convo.	0.244*	−0.093	0.074	**0.353** *
Social reputation	0.269*	0.066	0.017	**0.528** *
Life better/worse	−0.134*	−0.037	−0.059	**0.760** *
Consequences	−0.003	0.003	0.001	**0.869** *

*Note.* EFA = exploratory factor analysis. Factor loadings > |.30| are in bold type. *p < .05.

The four emergent factors were: (a) Daily Personal Impact (Factor 1, eight items), in which items describe the secret’s daily impact in terms of rumination frequency, effort invested in concealment, associated stress, and the need to make life adjustments, avoid social situations, and lie to others; (b) Relationship Impact (Factor 2, three items), in which items describe how keeping the secret affects relations with the target in terms of felt authenticity, perceived distance, and difficult interactions; (c) Pull to Reveal (Factor 3, three items), in which items describe the secret-keeper feeling external Pressure to Reveal the secret, as indicated by feelings of guilt, expectations from others, and obligations to disclose; and (d) Anticipated Consequences (Factor 4, four items), in which items describe the negative consequences expected upon revealing the secret, including uncomfortable conversations, social reputation damage, general negative consequences, and life becoming better or worse. As expected, these four factors distinguish among individual and social sources of secrecy burden. Factors 1 and 4, which assess Daily Personal Impacts and Anticipated Negative Consequences, are more self-oriented, whereas Factors 2 and 3, which assess relationship impacts and external pressures to reveal the secret, are more relationship-oriented. The emergence of these latter two factors as distinct also demonstrates that scale prompts did not dictate factor structure. Thus, our EFA lends support to our underlying hypothesis that secrecy burden consists of multiple dimensions, some of which stem from the secret-keeper’s relationship to the target.

#### Convergent & Discriminant Validity

Next, we wanted to compare this measure with prior assessments of secrecy burden. We computed participants’ average scores on each Secrecy Burden subscale using the corresponding factor items and participants’ overall burden scores. All subscales had acceptable or good reliability (Daily Personal Impact: α = .862, *M* = 3.72, *SD* = 1.28; Relationship Impact: α = .748, *M* = 4.36, *SD* = 1.22; Pressure to Reveal: α = .727, *M* = 3.34, *SD* = 1.66; Anticipated Consequences: α = .701, *M* = 4.72, *SD* = 1.27; Overall Burden: α = .831, *M* = 3.99, *SD* = 0.90). We then examined correlations with ruminative thought and the TSS-25 subscales (see Table 4).

**Table 4.** Zero-Order Correlations Among Secrecy Burden Subscales and Other Burden Measures.

Variable	Daily Personal Impact	Relationship Impact	Pressure to Reveal	Anticipated Consequences	Overall Burden
Ruminative thought	.500***	.351***	.187**	.092	.478***
TSS–disclosure apprehension	.333***	.128*	.257***	.686***	.531***
TSS–cognitive preoccupation	.654***	.290***	.343***	.183**	.638***

*Note.* TSS = Tilburg Secrecy Scale.

* *p* < .05. **p < .01. ***p < .001.

Overall, the new Secrecy Burden Scale did overlap with other measures, suggesting some convergent validity. However, the range of correlations across subscales suggests that prior measures were missing several key aspects of secrecy burden, thus bolstering our scale’s discriminant validity. For instance, both ruminative thought and the TSS-25 subscales had weak to moderate correlations with the Relationship Impact and Pull to Reveal subscales. On the other hand, Daily Personal Impact had the strongest correlations with these prior measures, which is unsurprising given that most items in this subscale overlapped with items from the other two measures. Overall, these data demonstrate that the new measure of secrecy burden still retains conventional indices of intrapersonal burden while also adding several crucial components of interpersonal burden sources.

### Discussion

Results of the EFA support a four-factor model: Daily Personal Impact, Relationship Impact, Pressure to Reveal, and Anticipated Consequences. The two factors of Daily Personal Impact and Anticipated Consequences focus on the individual secret-keeper (their daily experience with concealing the information and worrying about how revealing the secret will impact their life), whereas the two factors of Relationship Impact and Pressure to Reveal focus on more social aspects of concealment (obligations to the target and impacts on their relationship). Furthermore, we found evidence of both convergent and discriminant validity by comparing this measure with other self-report measures of secrecy burden. The two intrapersonal factors (Daily Personal Impact and Anticipated Consequences) were highly correlated with previously established measures. However, the two interpersonal factors (Relationship Impact and Pressure to Reveal) only had moderate to weak correlations. These latter two factors address how keeping a secret is more burdensome *because of* other relationships and social influences in the secret-keeper’s life. Thus, these results underscore the added value of this new measure in capturing the social components of secrecy burden that were neglected by prior measures.

## Study 2: Confirmatory Factor Analysis

The goal of Study 2 was to conduct a CFA using the observed factor structure from Study 1. We also wanted to test the measure’s predictive validity and included measures of personal well-being (i.e., flourishing, psychological distress), relational well-being (i.e., loneliness, authenticity), and personal or social resources that might alleviate secrecy burden or make it less detrimental to well-being (i.e., perceiving coping efficacy, perceived social support). These last two measures were selected from previous research on secrecy burden (see Slepian & Moulton-Tetlock, 2019), and the personal and relational well-being measures were selected from commonly-used assessments in the literature.

### Method

#### Participants

Participants were recruited via Prolific to complete a brief online study on how keeping a personal secret has impacted their lives. Sample size was based on the same factor analysis recommendations as Study 1 (Howard, 2016; Kyriazos, 2018; Widaman, 2012). Participants were excluded for not currently keeping a negative personal secret (*n =* 17). The final sample (*N* = 352, age range = 18-62) was 60.80% female and predominantly White (61.08%), followed by Black/African American (19.03%), Hispanic (8.52%), Asian/Pacific Islander (4.55%), other (3.41%), and Middle Eastern (0.57%). We again did not restrict countries and had a more global sample with similar countries as Study 1. Participants received U.S.$1.60 for completing the study.

#### Procedure

As in Study 1, participants had to currently be keeping a personal secret from at least one person. After reflecting on this secret, they completed the 18-item Secrecy Burden Scale, along with questions about how long they had been keeping the secret and how important, serious, and personal the information was. Participants then completed additional measures of perceived coping efficacy and social support for the secret (Slepian & Moulton-Tetlock, 2019), flourishing (Diener et al., 2010), psychological distress (Petrowski et al., 2019), loneliness (Hays & DiMatteo, 1987), authenticity (Wood et al., 2008), and attachment style (Fraley et al., 2011). Attachment style was not relevant to the current study and will not be discussed further.

#### Measures

##### Secrecy Burden

Participants completed the same 18-item Secrecy Burden Scale from Study 1. Welch two-sample t-tests on item means between Studies 1 and 2 showed no significant differences between sample means on any of the items (see the Supplemental material for all item means across Studies 1–3).

##### Perceived Coping Efficacy

Participants responded to three items assessing the extent that they felt capable in coping with their secret, in control over the situation, and were handling the secret well (1 = *not at all*, 7 = *very much*; Slepian & Moulton-Tetlock, 2019). Higher scores indicated higher coping efficacy for the secret (α = .739, *M* = 5.30, *SD* = 1.27).

##### Perceived Social Support

Participants responded to six items assessing the extent that they had received comfort, useful insights, emotional support, advice, and new perspectives from other people regarding the secret (1 = *not at all*, 7 = *very much*; Slepian & Moulton-Tetlock, 2019). Higher scores indicated higher social support for the secret (α = .917, *M* = 3.63, *SD* = 1.74).

##### Flourishing

Participants completed the eight-item Flourishing Scale (Diener et al., 2010). Example items include “My social relationships are supportive and rewarding” and “I am a good person and lead a good life” (1 = *strongly disagree*, 7 = *strongly agree*). This measure has been widely used to assess psychological well-being, with higher scores indicating higher psychological well-being (α = .901, *M* = 5.15, *SD* = 1.14).

##### Psychological Distress

Participants completed the Symptom Checklist K-9 (Petrowski et al., 2019), indicating the extent to which they experienced symptoms of psychological distress over the past week. Example items include “uncontrollable emotional outbursts” and “feeling uptight or agitated” (1 = *not at all*, 7 = *extremely*), with higher scores indicating higher levels of distress (α = .905, *M* = 3.46, *SD* = 1.74).

##### UCLA Loneliness Scale

Participants completed the eight-item UCLA Loneliness Scale (Hays & DiMatteo, 1987). Example items include “I lack companionship” and “I am an outgoing person” (reverse coded; 1 = *never*, 4 = *often*), with higher scores indicating more loneliness (α = .834, *M* = 2.38, *SD* = 0.65).

##### Inauthenticity

Participants responded to four items from the Self-Alienation subscale of Wood et al.’s (2008) Authenticity Scale. Example items include “I don’t know how I really feel inside” and “I feel out of touch with the ‘real me’” (1 = *strongly disagree*, 7 = *strongly agree*), with higher scores indicating greater feelings of inauthenticity (α = .892, *M* = 3.66, *SD* = 1.63).

#### Analysis Plan

As in Study 1, all analyses were conducted using the *MPlusAutomation* package in R (Hallquist & Wiley, 2018) to run models in Mplus 8.3 (Muthén & Muthén, 2017) through RStudio 4.1.2 (RStudio Team, 2021). Data were missing for eight items but did not exceed one or two missing responses per item, so that, we assume data were missing at random and did not perform any list-wise deletions.

CFA was conducted using maximum likelihood estimation with robust standard errors on the emergent factor structure from the EFA. Unit-loading identification was used to fix the reference variable for each latent factor to a factor loading of 1. For model fit evaluation, the same criteria were used as the EFA: a non-significant chi-square test, CFI = 0.90 to 0.95, RMSEA = 0.05 to 0.08, and SRMR 0.05 to 0.08 (Brown, 2015; Fabrigar et al., 1999; Hu & Bentler, 1999). Items were considered meaningful if the parameter estimate was statistically significant and above 0.30 (Brown, 2015). Modification indices were also requested to assess whether additional parameters would improve model fit. The parameter with the largest modification index (MI) was sequentially added to the model, provided that the parameter had a meaningful interpretation that could provide sensible model improvement (Brown, 2015). After adding each MI, the CFA was re-run and the next largest MI was added, repeating this process until the highest MI was no longer substantially different from the next MI and/or the parameter no longer provided a sensible addition to the model.

### Results

#### Confirmatory Factor Analysis

CFA was conducted to confirm the 18-item, four-factor structure that emerged from the EFA in Study 1. The overall fit indices for this four-factor model were: χ²(129) = 438.324, *p* < .001, RMSEA = 0.083 [0.074, 0.091], CFI = 0.837, and SRMR = 0.083. Given that most values were just outside our specified ranges for good model fit, modification indices were examined to improve model fit. Following our analysis plan, we sequentially added two modification indices: correlating anticipated consequences and life becoming better/worse upon revealing (StdYX EPC = 0.708; MOD1) and correlating avoiding social situations and daily life adjustments (StdYX EPC = 0.350; MOD2). After adding these parameters, the difference between the highest MI and subsequent indices was not substantial, and the suggested modifications did not conceptually add anything to the model, thus concluding our consideration of further MIs (see the Supplemental material for details).

**Figure 2.**
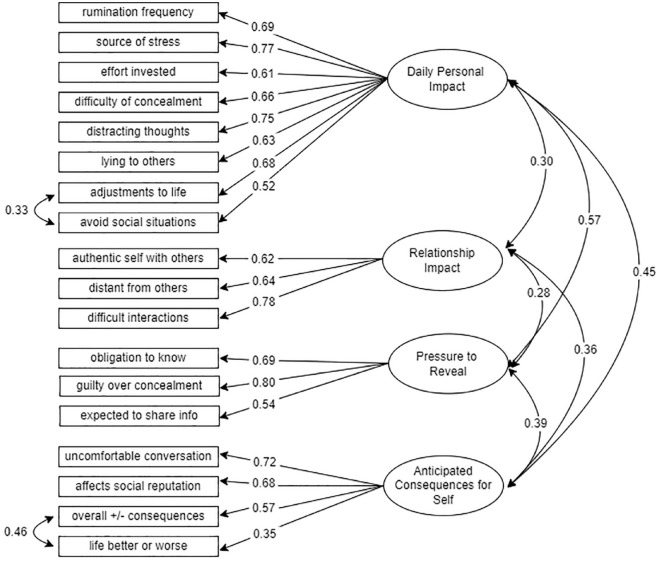
Path diagram of the final CFA Model with modification indices and standardized parameter estimates. *Note.* All paths were significant at *p* < .001.

Table 5 presents model fit statistics for all three models. The final model with both modification indices (CFA-MOD2) had the best fit indices, with RMSEA and SRMR now falling within our specified range of values for good model fit (0.05-0.08). The added modification indices improved model fit, and the added theoretical value made us confident in retaining those indices in the final model. Furthermore, all parameter estimates were statistically significant and above 0.30, with most estimates exceeding 0.50, thus indicating strong loadings (see Figure 2).

**Table 5.** Summary of Model Fit Indices From CFA With Modification Indices.

Model	Par	LL	χ^2^	*df*	*p* value	RMSEA [90% CI]	CFI	TLI	SRMR
CFA (initial)	60	−11,383.99	438.324	129	< .001	0.083 [0.074, 0.091]	0.837	0.806	0.083
CFA–MOD1	61	−11,356.58	378.499	128	< .001	0.075 [0.066, 0.083]	0.868	0.842	0.075
CFA–MOD2	62	−11,339.94	348.650	127	< .001	0.070 [0.062, 0.079]	0.883	0.859	0.074

*Note.* CFA = confirmatory factor analysis; MOD1 = model with one modification index; MOD2 = model with two modification indices; LL = log likelihood; RMSEA = root mean-square error of approximation; CI = confidence interval; CFI = comparative fit index; TLI = Tucker–Lewis index; SRMR = standardized root mean-square residual.

#### Zero-Order Correlations

To assess the measure’s predictive validity, we were interested in whether each subscale would be uniquely associated with well-being. We again computed participants’ average scores on each Secrecy Burden subscale using the corresponding factor items and on the overall measure.^3^ All subscales had acceptable or good reliability (Daily Personal Impact: α = .865, *M* = 3.67, *SD* = 1.28; Relationship Impact: α = .708, *M* = 4.35, *SD* = 1.11; Pressure to Reveal: α = .726, *M* = 3.35, *SD* = 1.58; Anticipated Consequences: α = .720, *M* = 4.66, *SD* = 1.21; Overall Burden: α = .849, *M* = 3.95, *SD* = 0.91). We then tested each subscale’s correlations with coping efficacy, perceived social support, flourishing, psychological distress, loneliness, and authenticity (see Table 6).

**Table 6.** Zero-Order Correlations Among Secrecy Burden Subscales and Well-Being Outcomes.

	Daily Personal Impact	Relationship Impact	Pressure to Reveal	Anticipated Consequences	Overall Burden
Psych. distress	.494***	.204***	.213***	.177***	.464***
Loneliness	.227***	.240***	−.083	.170***	.237***
Flourishing	−.082	–.177***	.031	–.132*	–.117*
Social support	.096*	−.099	.082	–.244***	−.008
Inauthenticity	.373***	.060	.196***	.123*	.338***
Coping efficacy	–.372***	–.331***	–.310***	–.106*	–.421***

* *p* < .05. ***p < .001.

Higher scores on overall burden and all burden subscales were associated with lower coping efficacy and greater psychological distress. All burden scores except for Pressure to Reveal were associated with greater loneliness. However, only Relationship Impact and Anticipated Consequences were associated with decreased flourishing. In addition, lower perceived social support for the secret was primarily associated with Anticipated Consequences. Interestingly, all burden scores except for Relationship Impact were associated with greater inauthenticity (despite one item in the Relationship Impact subscale specifically asking how authentic participants felt while keeping the secret). Thus, some well-being outcomes appeared to suffer consistently regardless of the burden source (but perhaps to varying degrees), whereas others were more uniquely associated with specific facets of secrecy burden.

### Discussion

Results from the CFA replicated the four-factor model from Study 1: Daily Personal Impact, Relationship Impact, Pressure to Reveal, and Anticipated Consequences. In addition, zero-order correlations suggest that well-being was differentially related to each burden subscale. Decreases in positive well-being (i.e., flourishing) were primarily associated with perceiving more harm to one’s relationships from keeping the secret and anticipating more negative consequences upon revealing it. That is, positive well-being appears to decrease when the secret’s negative implications extend beyond daily personal demands of keeping a secret and begin to implicate other areas of one’s life.

Furthermore, anticipating negative consequences upon revealing was associated with lower perceived social support. Participants with less social support may perceive their secret as having more negative implications given the lack of social reassurance to suggest otherwise, or alternatively, participants who think their secret will have particularly adverse consequences may perceive themselves as unable to reach out to others given the gravity of the secret. Finally, all aspects of secrecy burden were associated with more negative affect, decreased coping efficacy, and greater psychological distress, supporting previous findings on the negative personal consequences of keeping a secret.

## Study 3: Assessing Secrecy Burden and Well-Being Across Time

Our next goal was to use our multidimensional measure of secrecy burden to assess outcomes of keeping a negative personal secret over a brief time frame. Secret-keeping is a continuous process that involves repeated instances of mind-wandering and deciding whether to reveal the secret across various concealment contexts (Slepian, 2022). Accordingly, we expect that the ongoing burden of keeping a secret will contribute to negative well-being consequences across time. In addition, as Study 2 suggested that the burden factors were differentially related to personal and relational well-being outcomes, we wanted to extend those findings to assess how secrecy burden might predict changes in well-being across 2 weeks. We chose 2 weeks to minimize the likelihood that participants revealed their secrets during that time^4^ while also allowing for secrecy burden to accumulate across multiple instances of thinking about the secret or having to conceal it.

Study 3’s sample size, design, hypotheses, and analyses were preregistered through AsPredicted.org (https://aspredicted.org/1HL_ZTR). Assuming the information remained a secret over 2 weeks, we hypothesized that higher burden on all four factors at Time 1 (*T*1) would be associated with more anxiety and depression symptoms at Time 2 (*T*2). Based on the correlations from Study 2, we also hypothesized that higher burden on Relationship Impact and Anticipated Negative Consequences at *T*1 would be associated with decreased flourishing at *T*2. In addition, we expected higher burden on Relationship Impact and Pressure to Reveal at *T*1 to be associated with lower relationship satisfaction and intimacy at *T*2, as these two factors represent the social sources of burden. Finally, we expected higher burden on Daily Personal Impact and Pressure to Reveal to be associated with lower authenticity at *T*2. Although our primary hypotheses focus on the predictive powers of each subscale, we also report results for overall burden, as some researchers may be interested in a secret’s cumulative burden rather than its specific sources.

### Method

#### Participants

Participants were recruited via Prolific to complete a two-part study on how keeping a personal secret has impacted their lives. Participants received U.S.$3.50 for completing both parts (with partial compensation for only completing Part 1). Using the smallest significant correlation from Studies 1 and 2 (*r* = .23), an a priori power analysis in G*Power 3.1 (Faul et al., 2009) suggested that a sample size of *N* = 143 would detect an effect size of .23 with 80% power. However, we anticipated some participant attrition given the two-part nature of the study, so that, we aimed to recruit *N* = 200 at *T*1 to still have a sufficient sample size at *T*2. We also oversampled to account for participant exclusions if they were not currently keeping a personal secret (*n =* 20). The final *T*1 sample after exclusions (*N* = 209, age range = 18-61) was 47.84% female and predominantly White (51.20%), followed by Hispanic (33.49%), Black/African American (14.35%), Asian/Pacific Islander (1.44%), and Middle Eastern (0.49%). Participants’ current countries included (in descending order of frequency) Mexico, Portugal, South Africa, Poland, Chile, Italy, and the United Kingdom, among others.

Participants responded to the follow-up survey within 2 to 3.5 weeks after the first survey, with a total of *N* = 190 at *T*2. However, given our focus on the *ongoing* effects of secrecy burden, we excluded participants who had revealed their secret to the primary target since the first survey. Of the 190 respondents, 16 had fully revealed the secret to the target, and 15 reported some degree of revealing (i.e., revealing part of the secret but not all of it). Thus, our final sample size for *T*2 was *N* = 159.^5^ Exploratory analyses regarding the moderating effects of reveal status are in the Supplemental material.

#### Procedure

At *T*1, participants filled out basic demographics and the Fear of Negative Evaluation scale (Leary, 1983).^6^ They then indicated if they were currently keeping a personal secret about themselves from at least one person. After reflecting on the secret, participants provided a brief description and completed the 18-item Secrecy Burden Scale, along with other questions about how long they had been keeping the secret and how important, serious, and personal the information was. Participants then completed Slepian and Moulton-Tetlock’s (2019) measure of social support for the secret and indicated the one person from whom they most wished to conceal the information. This person was identified by initials, first names, or relationship labels (e.g., “best friend”), and these responses were piped into subsequent questions pertaining to the target. Participants then completed measures of authenticity with the target (Wood et al., 2008), relationship satisfaction (Rusbult et al., 1998), anxiety and depression symptoms (Derogatis & Melisaratos, 1983), flourishing (Diener et al., 2010), and a single-item measure of closeness.

Participants were contacted 2 weeks later to complete the follow-up survey. They began by recalling the personal secret and target they had reported at *T*1. They then indicated whether they had revealed the secret (in its entirety or partially) to the target or anyone else since the first survey. Afterwards, they completed the same measures of authenticity with the target, relationship satisfaction and closeness, anxiety and depression symptoms, and flourishing. Here, we focus on the individual and social well-being outcomes that were assessed at both time points. Analyses regarding fear of negative evaluation, perceived social support, and general secret characteristics can be found in the Supplemental material.

#### Measures

##### Secrecy Burden

Participants completed the same 18-item Secrecy Burden Scale from Studies 1 and 2 regarding a personal secret that they were currently keeping. For each participant, we computed an overall burden score (α = .850, *M* = 4.07, *SD* = 0.96) and individual scores on each subscale: Daily Personal Impact (α = .892, *M* = 3.73, *SD* = 1.41), Relationship Impact (α = .559, *M* = 4.48, *SD* = 1.07), Pressure to Reveal (α = .757, *M* = 3.46, *SD* = 1.73), and Anticipated Consequences (α = .719, *M* = 4.91, *SD* = 1.22). Correlations among subscales ranged from weak to moderate (*r* = .071, *p* = .308 to *r* = .423, *p* <.001).^7^

##### Inauthenticity

Participants completed the same four items from the Self-Alienation subscale of Authenticity (Wood et al., 2008) from Study 2 (*T*1: α = .890, *M* = 3.32, *SD* = 1.66; *T*2: α = .840, *M* = 3.24, *SD* = 1.43).

##### Relationship Satisfaction

Participants responded to five items from the Satisfaction subscale of the Investment Model Scale (Rusbult et al., 1998) regarding their relationship with the target. Example items include “I feel satisfied with our relationship” and “Our relationship is close to ideal” (1 = *strongly disagree*, 7 = *strongly agree*), with higher scores indicating greater relationship satisfaction (*T*1: α = .945, *M* = 4.80, *SD* = 1.64; *T*2: α = .960, *M* = 4.58, *SD* = 1.66). Participants also responded to the question “How close do you feel in your relationship with [Target]” using a 10-point slider scale to indicate overall feelings of closeness (*T*1: *M* = 7.01, *SD* = 2.74; T2: *M* = 6.47, *SD* = 2.87).

##### Anxiety/Depression Symptoms

Participants completed six items from the depression symptom dimension and six items from the anxiety symptom dimension of Derogatis and Melisaratos’ (1983) Brief Symptom Inventory. They were asked to indicate how much they had been bothered by each symptom over the past week (1 = *not at all*, *not at all*, 7 *= extremely*). Example items for anxiety include “feeling fearful” and “nervousness or shakiness inside.” Example items for depression include “feeling lonely” and “feelings of worthlessness.” Both scales had high reliability, with higher scores indicating greater symptom prevalence (anxiety, *T*1: α = .880, *M* = 3.38, *SD* = 1.59; anxiety, *T*2: α = .890, *M* = 3.20, *SD* = 1.52; depression, *T*1: α = .900, *M* = 4.01, *SD* = 1.69; depression, *T*2: α = .890, *M* = 3.70, *SD* = 1.70).

##### Flourishing

Participants completed the same eight-item Flourishing scale (Diener et al., 2010) from Study 2 (*T*1: α = .891, *M* = 4.96, *SD* = 1.17; *T*2: α = .910, *M* = 4.91, *SD* = 1.17).

### Results

#### Zero-Order Correlations at T1

To replicate and expand on the zero-order correlations from Study 2, we tested each subscale’s correlations with anxiety, depression, relationship satisfaction, flourishing, and authenticity at *T*1 and *T*2 (see Table 7). Similar to Study 2’s results regarding psychological distress, higher scores on all burden subscales and overall burden were correlated with greater symptoms of depression and anxiety. Furthermore, decreased flourishing was again associated with higher burden on the Relationship Impact and Anticipated Consequences subscales at *T*1.

**Table 7.** Zero-Order Correlations Among Secrecy Burden Subscales and Well-Being Outcomes.

Variable	Time Point	Daily Personal Impact	Relationship Impact	Pressure to Reveal	Anticipated Consequences	Overall Burden
Anxiety	*T*1	.506***	.214**	.300***	.138*	.500***
	*T*2	.350***	.336***	.257**	.303***	.449***
Depression	*T*1	.400***	.259***	.234***	.182**	.431***
	*T*2	.375***	.364***	.178*	.290***	.444***
Flourishing	*T*1	−.116	–.214**	.036	–.219**	–.166*
	*T*2	−.058	−.143	.132	–.168*	−.070
Inauthenticity	*T*1	.354***	.270***	.210**	.128	.403***
	*T*2	.369***	.312***	.239**	.269***	.440***
Relationship Satisfaction	*T*1	−.098	−.105	.029	.072	−.055
	*T*2	−.130	−.103	−.018	.067	−.091
Intimacy	*T*1	−.094	−.036	.064	.004	−.048
	*T*2	−.070	−.045	.001	.047	−.041

*Note.* Correlations for well-being outcomes between *T*1 and *T*2 ranged from *r* = .523 to *r* = .848.

* *p* < .05. **p < .01. *** *p* < .001.

However, contrary to Study 2 (but more consistent with theory-based expectations), feelings of inauthenticity were associated with higher burden on almost all subscales. Finally, and contrary to hypotheses, none of the burden subscales were associated with relationship satisfaction or closeness with the target. Thus, we replicated most zero-order correlation results from Study 2, except for relationship satisfaction and authenticity.

#### Predicting T2 Well-Being Outcomes From T1 Burden

Next, we conducted a series of multiple regression analyses to see whether participants’ secrecy burden on each subscale at *T*1 would predict their well-being outcomes at *T*2, controlling for *T*1 well-being. We conducted hierarchical regressions, entering participants’ *T*1 scores on the outcome variable as a predictor in Step 1 and adding the burden subscale or overall burden score as a second predictor in Step 2. Table 8 presents the results regarding participants’ *T*2 levels of anxiety, depression, and authenticity as predicted by each burden subscale and *T*1 levels of the corresponding outcome (with each line indicating a separate regression model).

**Table 8.** Predicting T2 Well-Being Outcomes From Burden Subscale Scores at T1.

Outcome & burden predictors	*b* [95% CI]	*t*	*p*
Anxiety			
Daily Personal Impact	0.06 [–0.09, 0.20]	*t*(156) = 0.79	.430
**Relationship Impact**	**0.28 [0.10, 0.46]**	*t* **(156) = 3.07**	.003**
Pressure to Reveal	0.07 [–0.04, 0.18]	*t*(156) = 1.23	.222
**Anticipated Consequences**	**0.22 [0.07, 0.37]**	*t* **(156) = 2.91**	.004**
**Overall Burden**	**0.27 [0.05, 0.48]**	*t* **(156) = 2.45**	.015*
Depression			
Daily Personal Impact	0.13 [–0.01, 0.26]	*t*(156) = 1.89	.061
**Relationship Impact**	**0.31 [0.13, 0.48]**	*t* **(156) = 3.47**	< .001***
Pressure to Reveal	0.05 [–0.05, 0.16]	*t*(156) = 0.98	.329
**Anticipated Consequences**	**0.18 [0.03, 0.33]**	*t* **(156) = 2.36**	.020*
**Overall Burden**	**0.30 [0.10, 0.50]**	*t* **(156) = 2.95**	.004**
Inauthenticity			
**Daily Personal Impact**	**0.23 [0.09, 0.36]**	*t* **(155) = 3.23**	.002**
**Relationship Impact**	**0.24 [0.05, 0.43]**	*t* **(155) = 2.46**	.015*
Pressure to Reveal	0.10 [–0.02, 0.22]	*t*(155) = 1.68	.095
**Anticipated Consequences**	**0.18 [0.02, 0.34]**	*t* **(155) = 2.21**	.029*
**Overall Burden**	**0.41 [0.20, 0.61]**	*t* **(155) = 3.87**	< .001***

*Note.* All analyses controlled for *T*1 levels of the well-being outcome (which were all significant). Each burden predictor was analyzed separately, with each line reporting the results of separate regression models.

* *p* < .05. **p < .01. ***p < .001.

##### Anxiety and Depression

For both anxiety and depression symptoms, higher scores on Relationship Impact, Anticipated Consequences, and Overall Burden at *T*1 were significantly associated with higher anxiety and depression symptoms at *T*2, controlling for *T*1 levels. Furthermore, although zero-order correlations showed that higher burden on Daily Personal Impact and Pressure to Reveal was significantly associated with greater anxiety and depression symptoms at *T*2, these effects no longer held after controlling for *T*1 symptoms. Thus, higher secrecy burden on the two subscales pertaining to broader implications of keeping the secret (Relationship Impact and Anticipated Consequences) and higher secrecy burden overall significantly predicted greater anxiety and depression symptoms 2 to 3 weeks later.

##### Inauthenticity

Higher scores on all burden subscales and overall burden at *T*1 predicted lower feelings of authenticity at *T*2, and all effects held when controlling for *T*1 authenticity except for Pressure to Reveal. Thus, nearly all facets of secrecy burden aside from external Pressure to Reveal predicted higher feelings of inauthenticity 2 to 3 weeks later.

##### Other Outcomes and Moderators

Consistent with the lack of significant correlations among relationship satisfaction, closeness, and burden subscales at *T*1 or *T*2, none of the regression analyses predicting relationship satisfaction or intimacy at *T*2 were significant, even controlling for *T*1 levels. Furthermore, after controlling for flourishing at *T*1, none of the burden subscales nor overall burden were significant predictors of flourishing at *T*2.

In addition, we re-ran all analyses controlling for general secret characteristics, specifically length, importance, and seriousness (see the Supplemental material). Briefly, the length of keeping the secret was not associated with any well-being outcomes or secrecy burden (except for Pressure to Reveal), and all effects were robust controlling for length. Thus, the effects of secrecy on well-being over 2 weeks were consistent regardless of whether the secret was relatively new or long-standing. However, there was evidence of importance and seriousness moderating the effects of Relationship Impact and Anticipated Consequences on authenticity at *T*2 (but not other well-being outcomes). Higher Relationship Impact burden was associated with less authenticity, but only for secrets of moderate or high importance and seriousness. In addition, when participants expected more negative consequences upon revealing, they felt less authentic for trivial secrets but not serious ones.

### Discussion

Study 3 showed that higher secrecy burden on most subscales was associated with higher depression, anxiety, and inauthenticity 2 to 3 weeks later, controlling for scores on those outcomes at *T*1. The one subscale that did not predict any well-being outcomes after controlling for *T*1 levels was Pressure to Reveal (discussed below). That is, although at a given moment external pressure was associated with more anxiety, depression, and inauthenticity, the continued negative impacts of secrecy burden appear to manifest primarily from higher burden on Relationship Impact, Anticipated Consequences, and Overall Burden. Daily Personal Impact similarly did not predict anxiety and depression symptoms after controlling for *T*1 levels, although higher burden on this subscale still predicted lower feelings of authenticity 2 to 3 weeks later. Thus, we see that secrecy burden in general is associated with lower well-being outcomes over time, but that different components of secrecy burden may vary in the extent to which they have momentary versus cumulative or lasting effects.

## General Discussion

This study provides the first attempt to develop a standardized measure of secrecy burden that accounts for both individual and social sources of burden. Results from the EFA and CFA support a four-factor model: Daily Personal Impact, Relationship Impact, Pressure to Reveal, and Anticipated Consequences. Daily Personal Impact is most comparable with how secrecy burden has been operationalized in past research, for it includes items assessing the effort and difficulty of concealment (cf. Bedrov & Leary, 2021) and rumination (cf. Slepian et al., 2016). However, these results also underscore the need to consider relational aspects of secrecy, as illustrated by the emergent factors of Relationship Impact and Pressure to Reveal, which assess the relationships with those from whom the secret is kept and subsequent social pressures. Moving forward, research on secrecy should aim to incorporate these multiple facets of secrecy burden instead of solely focusing on the cognitive or *intra-*personal toll. In addition, most correlations between factors were of moderate strength, suggesting that these factors are related yet distinct.

### Implications for Well-Being

Across Studies 2 and 3, each burden factor was differentially related to individual and relational well-being. For example, zero-order correlations suggested that decreased flourishing was primarily associated with how much the secret impacted relations with the target and could have adverse outcomes upon revealing. Although higher burden across all subscales was associated with greater negative outcomes, positive well-being only seemed to diminish when the secret had negative implications beyond Daily Personal Impacts. However, we did not see any longitudinal associations between secrecy burden and flourishing, suggesting that one secret alone might not be enough to change global evaluations of how meaningful or fulfilling one’s life is across multiple domains (Diener et al., 2010). Instead, thinking about a burdensome secret may momentarily lead to lower feelings of purpose or optimism, only to be recovered upon engaging with other aspects of one’s life. Indeed, these zero-order correlations might reflect momentary effects elicited by thinking about the secret in the context of the survey rather than how the secret was generally affecting participants’ well-being.

Similarly, feeling strong external Pressure to Reveal the secret was associated with current feelings of anxiety, depression, inauthenticity, and psychological distress but did not predict changes in these outcomes over time. If people feel obligated to reveal a secret to someone, they may experience momentary threats to autonomy. People believe in ownership over personal information and controlling who knows what under which circumstances (Petronio, 2016). Thinking about outside expectations to reveal a secret may manifest as a threat to one’s autonomy needs, which has been associated with lower well-being (e.g., Deci & Ryan, 2012). But, similar to flourishing, the perceived threat and negative outcomes might only arise when thinking about those autonomy threats in the context of the secret and then diminish once one exercises autonomy in other areas of life.

However, higher secrecy burden in terms of Daily Personal impact, Relationship Impact, and Anticipated Negative Consequences was associated with lower psychological well-being and authenticity over time. Thus, people seem to experience more adverse consequences across time when the secret starts to permeate their everyday routine and implicate their personal life and relationships. This finding underscores that secrecy burden is not only a combination of individual and social factors, but that its implications for well-being also depend on which aspects of keeping the secret are more or less burdensome.

### Theoretical Implications

Future research could benefit from this measure to investigate how secrecy burden and its consequences may be alleviated. For instance, researchers have distinguished between confessing a secret to the target and confiding it to a third party, with each action having different emotional and motivational antecedents (Nguyen & Slepian, 2022). However, it remains unclear whether one form of revealing the secret is more or less beneficial for relieving secrecy burden. Similarly, although we did not have a large enough subsample to address this question, it would be interesting to see whether partial revelations of a secret reap any well-being benefits. Examining changes in secrecy burden based on recipients’ perceived responsiveness can also add to existing research on how responses to self-disclosure and revealing secrets affect relationship quality, self-esteem, and rumination, among other outcomes. (e.g., Afifi & Caughlin, 2006; Kelly & McKillop, 1996; Laurenceau et al., 1998). This measure could also examine whether secrecy burden changes over time and progressively increases or diminishes the longer a secret is kept. Study 3 provided an initial attempt at examining secrecy burden over time, but a 2-week snippet is not sufficient for understanding the nature of secrecy burden at the very beginning of the concealment intention relative to several years later.

This measure can also be used with other approaches like latent profile analysis to examine how different patterns across the burden subscales correspond to different types of secrets or individuals. Secrets vary extensively in their content and severity, and this measure can serve as an important standardized tool for examining their relative consequences based on differing burden sources. Finally, this measure underscores the importance of considering social context and dyadic relationships. One person may keep the same secret from multiple people, but that does not mean that the secrecy burden will be the same in each relationship. Thus, it is important to consider how secrecy burden fluctuates not only across types of secrets but also across different relationships and individuals.

### Limitations and Future Directions

These studies do have their limitations, starting with an imbalance in item loading distributions across factors. Daily Personal Impact was defined by eight items, whereas Pull to Reveal and Relationship Impact were only defined by three items. The reliability of these latter two factors may be relatively weaker, which could be problematic for our purposes given that these two factors represent the more social sources of burden. Furthermore, although the factors are theoretically grouped into individual and social dimensions, certain items that were categorized as “individual” may still have strong social components. For example, Daily Personal Impact includes an item assessing the frequency of lying *to others* to keep the secret, and Anticipated Consequences includes an item on how one’s *social reputation* would be affected upon revealing. Both items consider interactions with other people, making them not entirely “individual.” More research is needed to better tease apart the distinctions between intra- and interpersonal burden. Given this limitation, we recommend the factors either be assessed separately as unique indices of different burden sources, or combined into one overall assessment of secrecy burden, regardless of its sources. The former approach would be of use to researchers interested in different *types* of secrecy burden (e.g., examining strategies to specifically reduce anticipated consequences burden), whereas the latter approach may be of use to researchers interested in the overall *level* of secrecy burden (e.g., examining well-being consequences of keeping secrets that are relatively higher or lower in overall burden).

Another key limitation is that participants did not describe their secrets in Studies 1 and 2, nor did we specify how serious or negative the secret needed to be. Descriptive statistics show that the secrets were quite personal, moderately serious, and slightly negative, and participants’ descriptions from Study 3 suggest that most secrets were significant rather than trivial, with common topics including infidelity, substance abuse, mental health, and sexual orientation. Nevertheless, we should exercise caution in discussing the practical applications of this scale without further examining secrecy burden in conjunction with the specific content.

Furthermore, we cannot make conclusions about the generalizability of this scale to different types of secrets. An important next step would be to assess measurement and structural invariance across different secrets (e.g., positive vs. negative, one’s own vs. someone else’s). Both the secret type and target may influence what is considered burdensome during concealment. For example, keeping someone else’s secret might not affect one’s social reputation but could create stress in terms of navigating who can or cannot know the information and how much agency one has in revealing the secret (see Petronio & Reierson, 2009, and other work on Communication Privacy Management Theory). In addition, secrecy burden may vary across cultures. Cultural differences in the emphasis on social harmony may be especially important when examining dimensions of secrecy burden and their correlates with well-being and relationship quality (e.g., Kim et al., 2006). In short, there is still much work to be done in examining secrecy burden across different contexts.

## Conclusion

These studies serve as an important step in measuring burden and bringing the interpersonal aspects of secrecy to the forefront. Our results show that secrecy burden entails multiple factors, some of which are relatively more individual or social. This study further highlights the benefits of using a standardized measure so as to better integrate multiple perspectives on secrecy burden and facilitate comparisons across studies. Finally, this research shows that secrecy burden is associated with well-being outcomes across time, emphasizing the practical importance of understanding the sources and extent of secrecy burden when concealing personal information.

## Supplemental Material

sj-docx-1-psp-10.1177_01461672231172387 – Supplemental material for How Much Is It Weighing on You? Development and Validation of the Secrecy Burden Scale Supplemental material, sj-docx-1-psp-10.1177_01461672231172387 for How Much Is It Weighing on You? Development and Validation of the Secrecy Burden Scale by Alisa Bedrov and Shelly L. Gable in Personality and Social Psychology Bulletin

## Notes

1. Recommendations for factor loading cutoffs typically fall between 0.30 and 0.40 (Bandalos & Finney, 2018; Brown, 2015; Howard, 2016). Since we already conducted two pilot studies to revise and finalize our items, we chose the lower cutoff value to retain as many variables as possible (Kahn, 2006).

2. The five-factor model split Daily Personal Impact into two factors, with the first encompassing cognitive personal burden (four items: ruminate, stress, difficulty, and distraction) and the second encompassing more action-based personal burden (four items: effort, life adjust, lying, avoid social).

3. We conducted a second-order CFA in Studies 2 and 3 to see whether the four factors could be combined into one overall score (see the Supplemental material). Briefly, Daily Personal Impact and Pressure to Reveal had high loadings on the second-order factor (0.60 and 0.72 in Study 2, 0.68 and 0.76 in Study 3), but Relationship Impact and Anticipated Consequences had lower loadings (0.35 and 0.45 in Study 2, 0.23 and 0.29 in Study 3; likely due to few items on the latter two subscales). However, the general reliability of the composite score and the fact that all parameter estimates still exceeded 0.30 make us relatively confident in using the Overall Burden score for some empirical questions.

4. Afifi and Caughlin (2006) found that across 2 months, 81.6% of participants did *not* reveal their secret, so that, we hoped that narrowing the timeline to 2 weeks would increase that proportion.

5. We also asked whether participants revealed the secret to anyone besides the target, completely (*n* = 21) or partially (*n* = 12), but did not exclude them from analyses since the information remained a secret from the primary target and our outcome measures concerned relations with the target.

6. A common reason for keeping secrets is to avoid negative evaluation (Caughlin et al., 2005), so that, we wanted to assess fear of negative evaluation as a potential individual difference that might moderate the effects of secrecy burden on well-being.

7. We ran another CFA using the 190 participants who completed *T*1 and *T*2, which confirmed the four-factor structure found in Studies 1 and 2 (see the Supplemental material).
