# Supplementary material for: How Much Is It Weighing on You? Development and Validation of the Secrecy Burden Scale
Source: Pers Soc Psychol Bull. 2023 May 24;50(9):1332–47. doi: 10.1177/01461672231172387 (PMC11318218; doi:10.1177/01461672231172387)
Supplement: sj-docx-1-psp-10.1177_01461672231172387 – Supplemental material for How Much Is It Weighing on You? Development and Validation of the Secrecy Burden Scale [file sj-docx-1-psp-10.1177_01461672231172387.docx]

**Supplemental Materials**

***Pilot Studies – Development of Scale Items***

**Cognitive Interview Process**

Pilot testing began with four cognitive interviews. Two interviewees were middle-aged adults, corresponding to the typical demographic of Amazon Mechanical Turk or Prolific participants, and two interviewees were college-aged students, corresponding to the typical demographic of participants recruited through university subject pools. The interview began with an open-ended prompt asking participants to think of a personal secret that they were currently keeping and describe their experience with the process of keeping that secret. After their initial response, participants were asked more pointed questions addressing aspects of secrecy burden that were commonly mentioned in the literature (e.g., the difficulty and effort of keeping the secret, distracting thoughts, effects on relationships). After responding to these questions, interviewees were asked to interpret the phrase “process of keeping the secret” and whether any questions were confusing, redundant, or left anything unaddressed regarding the secrecy process.

**Pilot Testing Items**

Based on the cognitive interviews and other research on secrecy burden, we created 18 items that addressed recurring themes from the experience of keeping a personal secret. Each item was assessed on a 7-point Likert scale, with items being grouped together based on initial leading prompts. In *Pilot Study 1a*, we recruited *N* = 484 participants through Prolific to think of a personal secret and respond to the corresponding scale items. We then conducted a principal component analysis with varimax rotation to see whether the items held together in meaningful factor groupings. Based on these results, the following items were *eliminated* for not having a primary loading above 0.30 and/or no cross-loadings above 0.30 (Brown, 2015):

- [When thinking about the people closest to you from whom you are keeping this secret…] To what extent do you wish you could reveal this secret to them right now?
- [When thinking about potentially revealing this secret to the people closest to you who don’t already know it…] How anxious do you feel?

For *Pilot Study 1b*, we created seven additional items to supplement the removed items so that the factors could still be defined by a sufficient number of items. We recruited *N* = 387 participants through Prolific to similarly think of a personal secret that they were currently keeping and respond to the corresponding scale items. We then conducted a principal component analysis with varimax rotation to see whether the items held together in any meaningful factor groupings. Based on the results, we eliminated the following items according to the same criteria as Pilot Study 1a:

*[When thinking about the people closest to you from whom you are keeping this secret…]*

- How often do you re-evaluate your decision to keep this information a secret from them?
- To what extent do you feel justified in keeping this secret from them?
- To what extent would you feel relieved if you were no longer keeping this secret from them?
- How much does keeping this secret prevent others from knowing the real you?
- To what extent has keeping this secret put a strain on your relationships with these people?

At this point, we had 18 items that grouped together into four meaningful factors, with each factor being defined by at least three items. Thus, we retained this final item set for all subsequent studies. The full list can be found below in the next section.

***Secrecy Burden Scale & Anchors***

*Prompt:* Everyone has secrets – information that they want to conceal from at least one person. It’s possible that certain other people already know this information, but you are trying to conceal the information from one or more people who do not already know it.

**Think of a personal secret** – a secret about you – that you are**currently** trying to keep from one or more people. We are not interested in the specific content of the secret and will not ask you to reveal any information about your secret in this study. We only ask that you think of a secret **about yourself,**not a secret that you know about somebody else.

Now that you have the secret in mind, please take a moment to **think about what influence keeping the secret has had on your life**. On the following pages, please select the responses that most accurately reflect your **current experience** with keeping this secret.

**Daily Personal Impact**

[Over the past week…]

*rumination –* How often did you think about the secret?

1 – not at all 4 – sometimes 7 – extremely often

*stress –* To what extent was the secret a source of stress in your everyday life?

1 – not at all 4 – somewhat 7 – extremely

*effort –* How much effort did you invest in keeping the secret?

1 – little to no effort 4 – some effort 7 – significant effort

*difficult –* How difficult was keeping the secret for you?

1 – not at all 4 – somewhat 7 – extremely

[Over the course of keeping this secret…]

*distraction –* How often have thoughts of this secret distracted you from daily activities?

1 – not at all 4 – sometimes 7 – extremely often

*life_adjust –* To what extent have you had to make adjustments in your daily life to conceal this information?

1 – not at all 4 – somewhat 7 – significantly

*lying –* How often have you had to lie to the people closest to you in order to keep this secret?

1 – not at all 4 – sometimes 7 – extremely often

[When thinking about the people closest to you from whom you are keeping this secret…]

*avoid_social –* How often have you had to avoid social situations because of this secret?

1 – not at all 4 – sometimes 7 – extremely often

**Anticipated Consequences**

[When thinking about potentially revealing this secret to the people closest to you who don’t know it…]

*uncomf_convo –* To what extent would revealing the secret be an uncomfortable conversation to have?

1 – not at all 4 – somewhat uncomfortable 7 – extremely uncomfortable

*social_reput –* Would revealing this secret negatively affect your social reputation/image?

1 – not at all 4 – somewhat negatively 7 – extremely negatively

*conseq_overall –* Overall, what type of consequences do you anticipate from revealing this information?

1 – extremely negative 4 – mixed; some negative some positive 7 – extremely positive

*life_betterworse –* To what extent do you anticipate your life becoming better or worse because you no longer were keeping this a secret?

1 – significantly worse 4 – mixed; some better some worse 7 – significantly better

[When thinking about the people closest to you from whom you are keeping this secret…]

**Pressure to Reveal**

*obligation -* To what extent do you think they have a right or an obligation to know this secret?

1 – not at all 4 – somewhat 7 – extremely

*guilt -* To what extent do you feel guilty about keeping this secret from them?

1 – not at all 4 – somewhat 7 – extremely

*expectations -* To what extent are you expected to share this type of information with them (i.e., the topic of your secret is something you would normally share with them)?

1 – not at all 4 – somewhat 7 – completely

**Relationship Impact**

*authenticity –* To what extent do you feel like your true authentic self when interacting with those people?

1 – not at all 4 – somewhat 7 – completely

*rel_distant –* To what extent has keeping this secret made you feel more distant or closer to them?

1 – significantly more distant 4 – no effect/neutral 7 – significantly closer

*interact_difficult –* To what extent has keeping this secret made your interactions with these people easier or more difficult?

1 – significantly more difficult 4 – no effect/neutral 7 – significantly easier

**Study 1 – Additional Measures, Tables, & Analyses**

**Measures**

**PANAS.** Participants responded to 28 items from the PANAS Scale (Watson & Clark, 1994) using a 5-point Likert scale to indicate how much they experienced each emotion over the past week (1 = *very slightly or not at all,* 5 = *extremely*). The items encompassed general positive and negative affect, as well as more specific emotions like guilt, sadness, and attentiveness (which were expected to be particularly relevant when keeping a secret). The 12 positive affect items were: alert, determined, enthusiastic, excited, attentive, calm, active, strong, relaxed, happy, joyful, and ease (α = .899, *M =* 2.73, *SD* = 0.86). The 16 negative affect items were: afraid, guilty, scared, irritable, nervous, ashamed, hostile, jittery, disgust, upset, distressed, self-dissatisfied, sad, blue, alone, and blameworthy (α = .936, *M =* 2.44, *SD* = 0.96).

**State Authenticity.** Participants responded to 3 items from Fleeson and Wilt’s (2010) measure of state authenticity, adapted to focus specifically on experiences while keeping the secret over the past week. The three items were: “How much were you acting like your true self while keeping this secret”, “How much were you putting on an act while keeping this secret?”, and “How accurate of an impression would someone have of you from the way you were acting while keeping this secret?” (1 = *not at all,* 7 = *very much*; α = .440, *M* = 4.60, *SD* = 1.26). The second item was reverse coded such that higher scores on the scale indicated higher state authenticity while keeping the secret. This measure had low reliability in our sample, given that the last two items were entirely uncorrelated with each other (*r* = .003, *p* = .961), despite being moderately correlated with the first item (item2: *r* = .303, *p* < .001; item3: *r* = .344, *p* < .001).

**Results**

***Data Screening & Descriptive Statistics***

Prior to analyses, each item was screened for violations of normality using density plots and skewness and kurtosis statistics. Based on the density plots, six items did not have a normal distribution. The item assessing how uncomfortable the conversation would be when revealing the secret was negatively skewed, with most respondents anticipating a highly uncomfortable conversation (*M* = 5.64, *SD* = 1.68). The item assessing how much revealing the secret would affect one’s social reputation had a bimodal distribution, with respondents either anticipating a minimal effect or significant effect. Additionally, items assessing how much participants had to avoid social situations, adjust their daily life, felt obligated to reveal the information, and were expected to share the information were all positively skewed. That is, a good deal of respondents reported generally low levels of avoiding social situations (*M* = 2.74, *SD* = 1.83), adjustments to daily life (*M* = 3.23, *SD* = 1.84), feelings of obligation (*M* = 3.01, *SD* = 2.06), and expectations to reveal the information (*M* = 3.31, *SD* = 1.98).

However, although not all density plots showed evidence of normal distributions, all skewness and kurtosis values were below |2.0| and |7.0| respectively, indicating no serious violations of normality (Curran et al., 1996). Furthermore, maximum likelihood estimation with robust standard errors is robust to slight violations of normality, so we did not perform any data transformations on our items.

Table 1S presents all bivariate correlations among the 18 items of the Secrecy Burden Scale. Table 2S below presents the zero-order correlations among each subscale, overall burden, and the additional measures of positive and negative affect and state authenticity. Higher scores on all burden subscales and overall burden were associated with more negative affect and lower feelings of state authenticity. However, higher burden in terms of Daily Personal Impact and Pressure to Reveal was not associated with less positive affect. Instead, overall burden and the two subscales pertaining to the broader implications of keeping a secret (Relationship Impact and Anticipated Consequences) were associated with lower positive affect. These later correlations are consistent with our primary findings from Study 2, in which overall burden and these two subscales were associated with lower flourishing. Thus, the negative implications of keeping a burdensome secret seemed to partly depend on which aspect of secrecy burden was particularly high.

Table 2S

*Zero-Order Correlations Among Secrecy Burden Subscales and Additional Measures*

|  | Daily Personal Impact | Relationship Impact | Pressure to Reveal | Anticipated Consequences | Overall Burden | |
| --- | --- | --- | --- | --- | --- | --- |
| PANAS – neg. | **.441***** | **.248***** | **.265***** | **.230***** | | **.486***** |
| PANAS – pos. | –.092 | **–.221***** | .004 | **–.134*** | | **–.147*** |
| State Authen. | **–.324***** | **–.343***** | **–.150**** | **–.119*** | | **–.363***** |

*Note.* **p* < .05, ***p* < .01, ****p* < .001.

Table 1S

*Inter-item correlations with confidence intervals for the 18-item Secrecy Burden Scale (Study 1)*

| Var. | | | 1 | 2 | 3 | 4 | 5 | 6 | 7 | 8 | 9 | 10 | 11 | 12 | 13 | 14 | 15 | 16 | 17 |
| --- | --- | --- | --- | --- | --- | --- | --- | --- | --- | --- | --- | --- | --- | --- | --- | --- | --- | --- | --- |
|  |  |  |  |  |  |  |  |  |  |  |  |  |  |  |  |  |  |  |  |
| 1. ruminate | | |  |  |  |  |  |  |  |  |  |  |  |  |  |  |  |  |  |
|  |  |  |  |  |  |  |  |  |  |  |  |  |  |  |  |  |  |  |  |
| 2. stress---- | | | .57** |  |  |  |  |  |  |  |  |  |  |  |  |  |  |  |  |
|  |  |  | [.49, .64] |  |  |  |  |  |  |  |  |  |  |  |  |  |  |  |  |
|  |  |  |  |  |  |  |  |  |  |  |  |  |  |  |  |  |  |  |  |
| 3. effort---- | | | .39** | .41** |  |  |  |  |  |  |  |  |  |  |  |  |  |  |  |
|  |  |  | [.29, .48] | [.31, .50] |  |  |  |  |  |  |  |  |  |  |  |  |  |  |  |
|  |  |  |  |  |  |  |  |  |  |  |  |  |  |  |  |  |  |  |  |
| 4. difficulty | | | .44** | .51** | .50** |  |  |  |  |  |  |  |  |  |  |  |  |  |  |
|  |  |  | [.34, .52] | [.42, .59] | [.41, .58] |  |  |  |  |  |  |  |  |  |  |  |  |  |  |
|  |  |  |  |  |  |  |  |  |  |  |  |  |  |  |  |  |  |  |  |
| 5. distraction | | | .56** | .61** | .40** | .50** |  |  |  |  |  |  |  |  |  |  |  |  |  |
|  |  |  | [.48, .63] | [.53, .67] | [.30, .49] | [.41, .58] |  |  |  |  |  |  |  |  |  |  |  |  |  |
|  |  |  |  |  |  |  |  |  |  |  |  |  |  |  |  |  |  |  |  |
| 6. life adjust | | | .43** | .49** | .47** | .40** | .50** |  |  |  |  |  |  |  |  |  |  |  |  |
|  |  |  | [.33, .52] | [.40, .57] | [.38, .56] | [.30, .49] | [.41, .58] |  |  |  |  |  |  |  |  |  |  |  |  |
|  |  |  |  |  |  |  |  |  |  |  |  |  |  |  |  |  |  |  |  |
| 7. lying- -- | | | .28** | .36** | .49** | .41** | .32** | .49** |  |  |  |  |  |  |  |  |  |  |  |
|  |  |  | [.17, .38] | [.26, .45] | [.40, .57] | [.31, .50] | [.21, .42] | [.40, .58] |  |  |  |  |  |  |  |  |  |  |  |
|  |  |  |  |  |  |  |  |  |  |  |  |  |  |  |  |  |  |  |  |
| 8. avoid----- ----social | | | .34** | .37** | .40** | .37** | .39** | .52** | .44** |  |  |  |  |  |  |  |  |  |  |
|  |  |  | [.24, .44] | [.27, .47] | [.30, .49] | [.26, .46] | [.29, .48] | [.43, .60] | [.34, .53] |  |  |  |  |  |  |  |  |  |  |
|  |  |  |  |  |  |  |  |  |  |  |  |  |  |  |  |  |  |  |  |
| 9. distant-- | | | -.17** | -.24** | -.08 | -.09 | -.20** | -.20** | -.09 | -.22** |  |  |  |  |  |  |  |  |  |
|  |  |  | [-.28, -.06] | [-.34, -.13] | [-.19, .04] | [-.20, .02] | [-.30, -.09] | [-.30, -.09] | [-.20, .03] | [-.33, -.11] |  |  |  |  |  |  |  |  |  |
|  |  |  |  |  |  |  |  |  |  |  |  |  |  |  |  |  |  |  |  |
| 10. interact difficult | | | -.16** | -.24** | -.15* | -.13* | -.23** | -.18** | -.18** | -.27** | .72** |  |  |  |  |  |  |  |  |
|  |  |  | [-.27, -.05] | [-.34, -.13] | [-.25, -.03] | [-.24, -.02] | [-.33, -.12] | [-.29, -.07] | [-.29, -.07] | [-.37, -.16] | [.67, .77] |  |  |  |  |  |  |  |  |
|  |  |  |  |  |  |  |  |  |  |  |  |  |  |  |  |  |  |  |  |
| 11. authentic | | | -.21** | -.18** | -.13* | -.16** | -.25** | -.22** | -.21** | -.24** | .40** | .46** |  |  |  |  |  |  |  |
|  |  |  | [-.32, -.10] | [-.29, -.07] | [-.24, -.02] | [-.27, -.05] | [-.36, -.14] | [-.33, -.11] | [-.31, -.10] | [-.34, -.13] | [.30, .49] | [.36, .54] |  |  |  |  |  |  |  |
|  |  |  |  |  |  |  |  |  |  |  |  |  |  |  |  |  |  |  |  |
| 12. obligation | | | .15* | .17** | .17** | .31** | .24** | .16** | .24** | .20** | -.01 | -.04 | -.08 |  |  |  |  |  |  |
|  |  |  | [.03, .26] | [.06, .28] | [.06, .28] | [.20, .41] | [.13, .35] | [.04, .26] | [.13, .34] | [.09, .31] | [-.12, .10] | [-.15, .08] | [-.20, .03] |  |  |  |  |  |  |
|  |  |  |  |  |  |  |  |  |  |  |  |  |  |  |  |  |  |  |  |
| 13. expectation | | | .22** | .23** | .16** | .27** | .17** | .25** | .19** | .17** | -.01 | -.04 | .00 | .48** |  |  |  |  |  |
|  |  |  | [.10, .32] | [.12, .34] | [.05, .27] | [.16, .37] | [.06, .28] | [.14, .35] | [.07, .29] | [.06, .28] | [-.13, .10] | [-.15, .07] | [-.11, .11] | [.39, .56] |  |  |  |  |  |
|  |  |  |  |  |  |  |  |  |  |  |  |  |  |  |  |  |  |  |  |
| 14. guilt---- | | | .33** | .36** | .38** | .42** | .39** | .23** | .41** | .28** | -.13* | -.21** | -.20** | .58** | .35** |  |  |  |  |
|  |  |  | [.23, .43] | [.26, .45] | [.28, .47] | [.32, .51] | [.29, .48] | [.12, .33] | [.31, .50] | [.17, .38] | [-.24, -.01] | [-.32, -.10] | [-.30, -.08] | [.50, .65] | [.25, .45] |  |  |  |  |
|  |  |  |  |  |  |  |  |  |  |  |  |  |  |  |  |  |  |  |  |
| 15. uncomf convo | | | .19** | .21** | .24** | .16** | .21** | .17** | .16** | .14* | -.18** | -.19** | -.18** | .13* | -.04 | .28** |  |  |  |
|  |  |  | [.08, .29] | [.10, .32] | [.13, .34] | [.05, .27] | [.10, .31] | [.06, .28] | [.05, .27] | [.03, .25] | [-.29, -.07] | [-.30, -.08] | [-.29, -.07] | [.02, .24] | [-.15, .07] | [.17, .38] |  |  |  |
|  |  |  |  |  |  |  |  |  |  |  |  |  |  |  |  |  |  |  |  |
| 16. social reputation | | | .09 | .11 | .23** | .07 | .15* | .21** | .25** | .19** | -.09 | -.04 | -.14* | .08 | -.04 | .23** | .40** |  |  |
|  |  |  | [-.03, .20] | [-.01, .22] | [.12, .33] | [-.04, .18] | [.03, .26] | [.10, .32] | [.14, .35] | [.08, .29] | [-.21, .02] | [-.16, .07] | [-.25, -.03] | [-.04, .19] | [-.15, .08] | [.12, .33] | [.30, .49] |  |  |
|  |  |  |  |  |  |  |  |  |  |  |  |  |  |  |  |  |  |  |  |
| 17. life better/worse | | | -.15** | -.14* | -.02 | -.12* | -.16** | -.09 | -.01 | -.17** | -.04 | -.09 | -.09 | -.10 | -.16** | -.03 | .18** | .35** |  |
|  |  |  | [-.26, -.04] | [-.25, -.03] | [-.13, .09] | [-.23, -.01] | [-.27, -.05] | [-.20, .03] | [-.12, .10] | [-.28, -.06] | [-.15, .07] | [-.20, .02] | [-.20, .03] | [-.22, .01] | [-.27, -.05] | [-.14, .09] | [.07, .29] | [.25, .45] |  |
|  |  |  |  |  |  |  |  |  |  |  |  |  |  |  |  |  |  |  |  |
| 18. consequence | | | -.08 | -.06 | .09 | .01 | -.08 | .06 | .09 | -.07 | -.07 | -.07 | -.15** | -.01 | -.10 | .11 | .30** | .42** | .68** |
|  |  |  | [-.19, .03] | [-.17, .05] | [-.02, .20] | [-.10, .12] | [-.20, .03] | [-.05, .17] | [-.03, .20] | [-.18, .04] | [-.18, .05] | [-.18, .04] | [-.26, -.04] | [-.13, .10] | [-.21, .01] | [-.00, .22] | [.19, .40] | [.32, .51] | [.61, .73] |
|  |  |  |  |  |  |  |  |  |  |  |  |  |  |  |  |  |  |  |  |

*Note.* Values in square brackets indicate the 95% confidence interval for each correlation, i.e., the plausible range of population correlations that could have caused the sample correlation (Cumming, 2014). **p* < .05. ***p* < .01.

**Study 2 – Additional Analyses**

**Results**

***Confirmatory Factor Analysis***

CFA was conducted to confirm the 18-item, four-factor structure that emerged from the EFA in Study 1. The overall fit indices for this four-factor model were: χ²(129) = 438.324, *p* < .001, RMSEA = .083 [.074 .091], CFI = .837, and SRMR = .083. Given that most values were just outside our specified ranges for good model fit, modification indices were examined to try to improve model fit.

The first modification index that we considered was to correlate the items assessing anticipated consequences upon revealing the secret and whether one’s life would become better or worse (StdYX EPC = .708). Conceptually, these two items ought to be highly correlated, as both assess the overall valence of life outcomes upon revealing. After adding this parameter to the model, we re-ran the modification indices and considered the next highest MI, which was to correlate the items assessing avoiding social situations due to keeping the secret and adjustments made to everyday life (StdYX EPC = 0.350). Correlating these two items also made conceptual sense, as both items assess changes to one’s normal routine in order to keep the secret, with avoiding social situations being one of those life adjustments. After adding this parameter to the model, we re-ran the modification indices, at which point the difference between the highest MI and subsequent indices was not substantial, and the suggested modifications did not conceptually add anything to the model. Thus, we stopped adding new parameters to the model.

Table 3S presents the model fit statistics for all three models. The final model with both modification indices had the best fit indices, with the RMSEA and SRMR now falling within our specified range of values for good model fit (0.05-0.08). The added modification indices improved model fit, and the added theoretical value made us confident in retaining those indices in the final model. Furthermore, all parameter estimates were statistically significant and above 0.30, with most estimates exceeding 0.50, thus indicating a strong loading (see Figure 2).

Table 3S


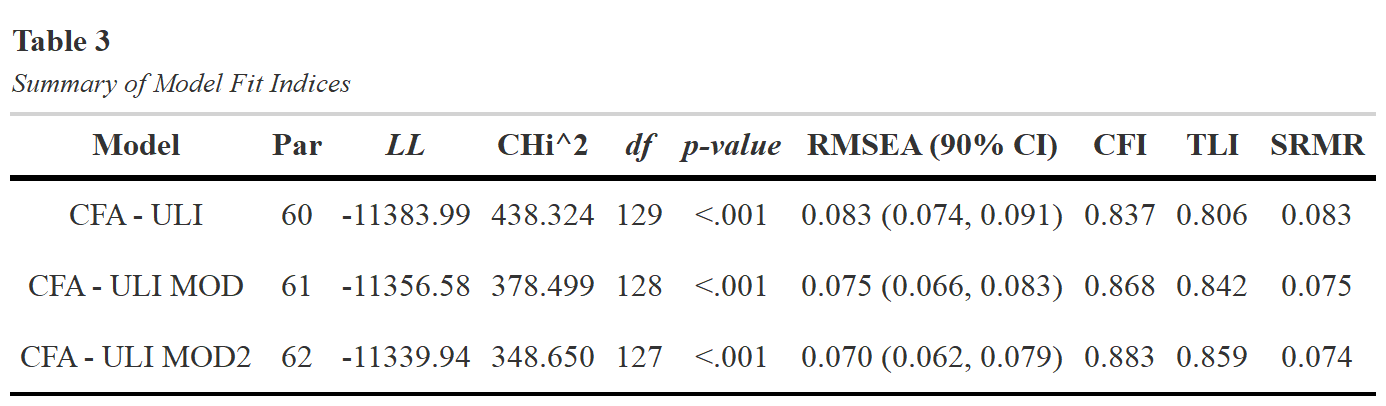
*Summary of Model Fit Indices from CFA with Modification Indices*

***Second-Order Factor Analysis***

Given that some researchers may be interested in the overall level of secrecy burden (regardless of its different facets), we computed participants’ average scores on overall burden using all 18 items. This composite score had good reliability (α = .849, *M* = 3.95, *SD* = 0.91). However, we wanted to further test whether a second-order confirmatory factor analysis would support combining all four subscales into a single composite score. Thus, we re-ran our original CFA model with an additional parameter of all four subscales loading onto a single second-order factor that represented Overall Burden. The overall fit indices for this second-order model were: χ²(135) = 459.587, *p* < .001, RMSEA = .083 [.074 .091], CFI = .829, and SRMR = .098. Figure 1S shows the parameter estimates for all items and subscales in this model.

The model fit statistics fell just outside our specified ranges for good model fit. Furthermore, based on parameter estimates, Relationship Impact and Anticipated Consequences both had relatively weak loadings on the second-order factor. It is important to note that both factors are only defined by three items rather than four or eight, thus reducing their reliability. Additionally, from a theoretical standpoint, both factors represent more situational aspects of secrecy burden that might not necessarily be present for all secrets or across all individuals. Specifically, the items in Daily Personal Impact and Pressure to Reveal represent relatively consistent aspects of secrecy burden that can be present in everyday life. For example, a secret-keeper may ruminate over their secret or feel guilty about not revealing the secret at any point. However, Relationship Impact is primarily salient when the secret-keeper interacts with or thinks about the target (which may vary drastically across individuals), and Anticipated Consequences is likely more salient when the secret-keeper considers the possibility of revealing their secret (which might not even be a consideration depending on circumstances). Thus, although not all four factors had strong loadings on the higher-order factor of Overall Burden, the general reliability of the composite score and the fact that all parameter estimates still exceeded 0.30 make us relatively confident in using the overall burden score for some empirical questions.

**Figure 1S**

*Path diagram of the second-order CFA model from Study 2 with standardized parameter estimates. All paths were significant at p < .001.*


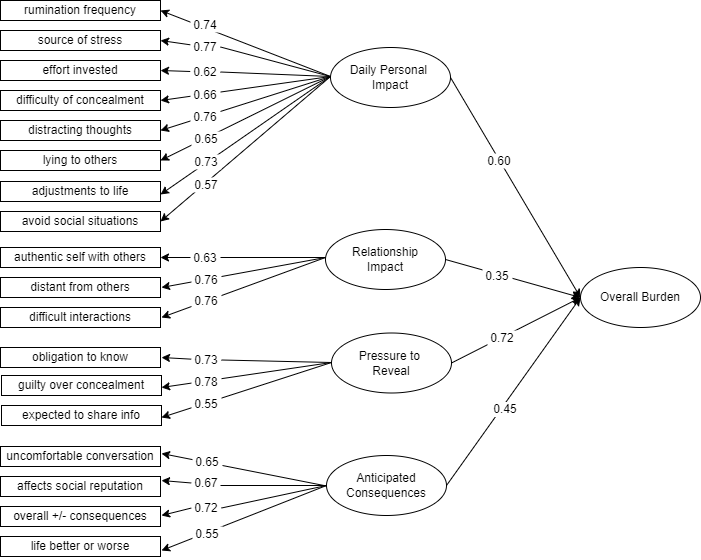


**Study 3 – Additional Measure, Tables, & Analyses**

**Measures**

**Secret Characteristics.** Participants responded to four additional items assessing how important it was that the information remain a secret (*M* = 5.04, *SD* = 1.81), how serious the information was (*M* = 4.53, *SD* = 1.92), how personal the information was (*M* = 6.13, *SD* = 1.35), and whether the information was good or bad from their perspective (*M* = 2.60, *SD* = 1.40). All responses were on 7-point Likert scales (1 = *not at all,* 7 = *extremely* or 1 = *very bad,* 7 = *very good*)*.* Participants also indicated how long they had been keeping the secret (*M* = 3.33 years, *SD* = 4.29 years; range: 3 days to 30 years; median: 2 years).

**Fear of Negative Evaluation.** Participants completed the 12-item Fear of Negative Evaluation Scale (FNE; Leary, 1983). Example items include “I am usually worried about what kind of impression I make” and “I often worry that I will say or do the wrong things” (1 = *not at all characteristic of me,* 7 = *extremely characteristic of me*), with higher scores indicating greater concerns with social evaluations (α = .737, *M* = 3.05, *SD* = 0.60).

**Perceived Social Support.** Participants completed the same 6-item social support scale (Slepian & Moulton-Tetlock, 2019) from Study 2 assessing the extent to which they had received comfort, useful insights, emotional support, advice, and new perspectives from other people regarding the secret (1 = *not at all,* 7 = *very much;* α = .949, *M* = 3.46, *SD* = 1.92).

**Results**

Table 4S presents all item means and standard deviations for each burden scale item across all three studies.

**Table 4S**

| **Item Name** | **Study 1** | **Study 2** | **Study 3** |
| --- | --- | --- | --- |
| 1. ruminate | 4.36 (1.65) | 4.43 (1.55) | 4.21 (1.79) |
| 2. stress | 3.86 (1.74) | 3.81 (1.76) | 3.65 (1.90) |
| 3. effort | 4.54 (1.82) | 4.37 (1.89) | 4.48 (1.82) |
| 4. difficulty | 3.54 (1.85) | 3.63 (1.89) | 3.86 (1.93) |
| 5. distraction | 3.41 (1.65) | 3.35 (1.73) | 3.37 (1.79) |
| 6. life adjust | 3.23 (1.84) | 3.16 (1.81) | 3.25 (1.85) |
| 7. lying | 4.11 (1.92) | 3.99 (1.84) | 4.41 (1.92) |
| 8. avoid social | 2.74 (1.83) | 2.59 (1.78) | 2.86 (1.93) |
| 9. distant ^a^ | 4.77 (1.41) | 4.66 (1.29) | 4.91 (1.32) |
| 10. interact difficult ^a^ | 4.75 (1.28) | 4.64 (1.23) | 4.85 (1.25) |
| 11. authentic ^a^ | 3.58 (1.75) | 3.76 (1.61) | 3.67 (1.77) |
| 12. obligation | 3.01 (2.06) | 3.06 (1.94) | 3.32 (2.12) |
| 13. expectations | 3.31 (1.98) | 3.22 (1.87) | 3.16 (2.01) |
| 14. guilt | 3.70 (2.14) | 3.77 (2.08) | 3.89 (2.21) |
| 15. uncomf convo | 5.64 (1.68) | 5.48 (1.66) | 5.81 (1.61) |
| 16. social reputation | 4.07 (2.21) | 3.99 (2.01) | 4.36 (2.10) |
| 17. life better/worse ^a^ | 4.30 (1.56) | 4.28 (1.43) | 4.35 (1.50) |
| 18. consequences ^a^ | 4.88 (1.50) | 4.89 (1.40) | 5.13 (1.31) |

*Item Means and Standard Deviations for Secrecy Burden Scale (All Studies)*

^a^ Indicates reverse-coded items. Item means were computed after reverse coding.

***Confirmatory Factor Analysis***

CFA was conducted to confirm the 18-item, four-factor structure that emerged in both Studies 1 and 2 using the 190 participants who provided data at both T1 and T2. The overall fit indices for this four-factor model were: χ²(129) = 297.247, *p* < .001, RMSEA = .083 [.071 .095], CFI = .852, and SRMR = .092. Given that most values were just outside our specified ranges for good model fit, modification indices were examined to try to improve model fit.

Similar to Study 2, the first modification index was to correlate the items assessing the anticipated consequences upon revealing the secret and whether one’s life would become better or worse (StdYX EPC = 2.301). Adding this parameter improved model fit such that model fit criterion now fell within our specified ranges (see Table 5S). Thus, we stopped adding new parameters to the model. All parameter estimates were statistically significant and above 0.30, with most estimates exceeding 0.50, thus indicating a strong loading (see Figure 2S). All factor loadings were also of similar degrees as those in Study 2, except for the items that loaded onto the Relationship Impact factor, which were relatively weaker but still exceeded 0.50.

**Table 5S**


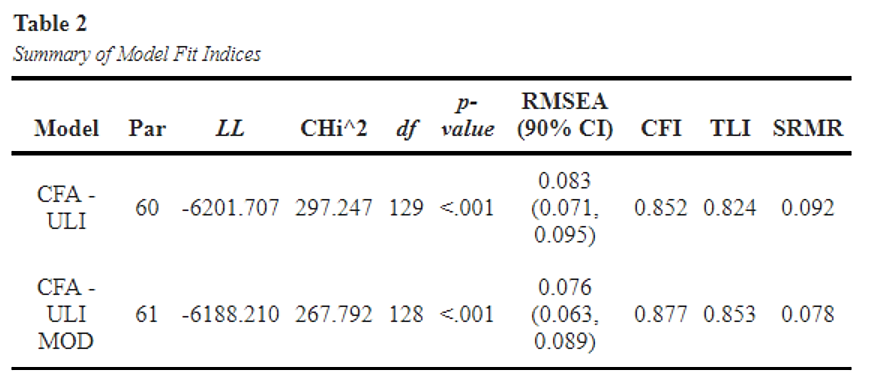
*Summary of Model Fit Indices from CFA with Modification Indices*

**Figure 2S**

*Path diagram of the final CFA model with modification indices and standardized parameter estimates. All paths were significant at p < .001.*


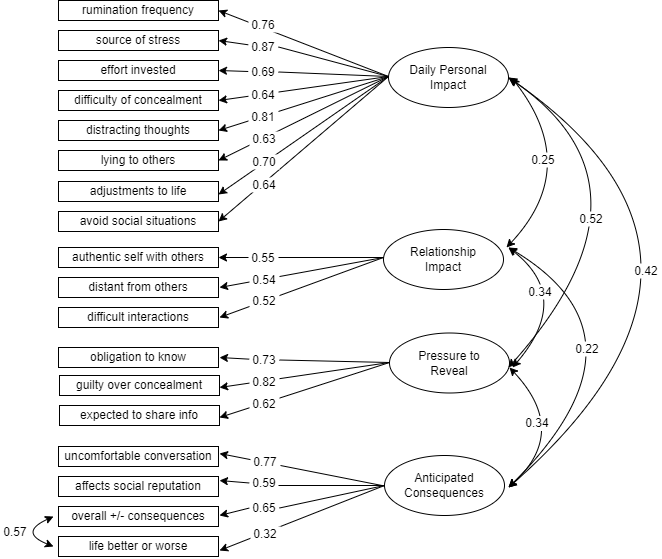


***Second-Order Factor Analysis***

As in Study 2, we computed participants’ average scores on overall burden using all 18 items. This composite score had good reliability (α = .850, *M* = 4.07, *SD* = 0.96). Next, we re-ran our original CFA model with an additional parameter of all four subscales loading onto a single second-order factor that represented Overall Burden. The overall fit indices for this second-order model were: χ²(135) = 294.179, *p* < .001, RMSEA = .079 [.0767 .091], CFI = .860, and SRMR = .097. Figure 3S shows the parameter estimates for all items and subscales in this model.

The model fit statistics fell just outside our specified ranges for good model fit (aside from RMSEA, which did suggest adequate fit). Furthermore, based on parameter estimates, Relationship Impact and Anticipated Consequences once again had relatively weak loadings on the second-order factor, this time falling below the threshold of 0.30. Similar to Study 2, we believe that these lower parameter estimates may reflect the aspects of secrecy burden that are less universally salient across secret-keepers. Still, the composite score showed good reliability, and a comprehensive assessment of secrecy burden would not be complete without consideration of the secret’s relational impact and potential consequences upon revealing. However, given the consistently low factor loadings for Relationship Impact and Anticipated Consequences onto the second-order factor of Overall Burden, we may recommend that researchers consider whether their research questions benefit from separating the scales rather than using a composite score.

**Figure 3S**

*Path diagram of the second-order CFA model from Study 3 with standardized parameter estimates. All paths were significant at p < .001.*


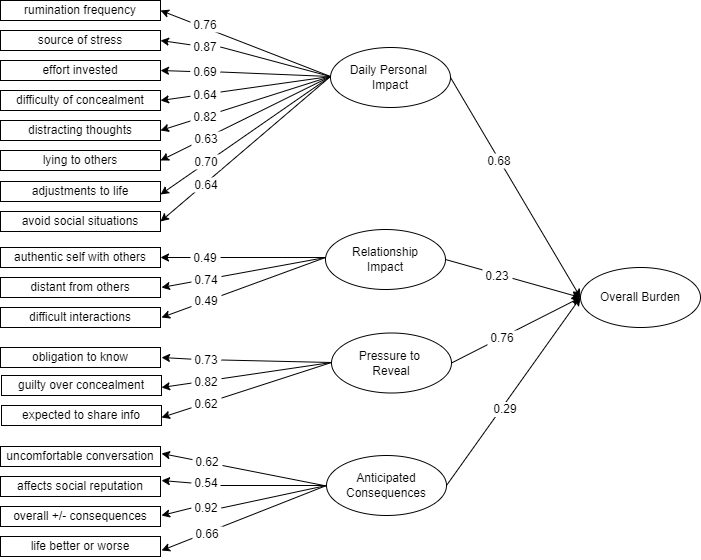


**Potential Moderators: Individual Differences.** We hypothesized two potential moderators of the effects, fear of negative evaluation and perceived social support. Fear of negative evaluation addresses intrapersonal concerns regarding sharing personal information, whereas perceived social support addresses the interpersonal resources one could rely on when dealing with a burdensome secret. Table 6S presents the zero-order correlations among each burden subscale and these two moderators. Table 7S presents the correlations among the two moderators and our primary well-being outcomes. Perceived social support was not significantly correlated with most outcome measures, and it was only associated with higher burden on the Anticipated Consequences subscale (similar to Study 2). Given the lack of significant correlations, we did not conduct any follow-up regression analyses with this moderator.

**Table 6S**

*Zero-Order Correlations Among Burden Subscales and Potential Moderators at T1*

|  | Daily Personal Impact | Relationship Impact | | Pressure to Reveal | Anticipated Consequences | Overall Burden |
| --- | --- | --- | --- | --- | --- | --- |
| Social Support | .039 | –.026 | .059 | | **–.194**** | –.016 |
| Fear of Negative  Evaluation | **.195**** | **.137*** | .106 | | **.140*** | **.224**** |

*Note. *p* < .05, ***p* < .01, *** *p* < .001.

Fear of negative evaluation, however, was significantly associated with almost all outcomes aside from relationship satisfaction. Furthermore, it was associated with higher perceived burden on Daily Personal Impact, Relationship Impact, Anticipated Consequences, and overall burden. Thus, we conducted a series of regression analyses in which we included the interaction between fear of negative evaluation and each burden subscale in predicting the subsequent outcomes while controlling for T1 levels of the outcome. That is, we wanted to see if the negative effects of secrecy burden differed among people who were higher or lower in fear of negative evaluation. For anxiety, depression, and authenticity, none of the interaction terms were significant, and the only significant predictors in each regression model were T1 levels of the well-being outcome. However, fear of negative evaluation did have some significant and marginally significant interactions with burden subscales when predicting flourishing at T2, most prominently with overall burden and marginally with Pressure to Reveal (see Table 8S).

**Table 7S**

|  | Social Support | Fear of Negative Evaluation |
| --- | --- | --- |
| Anxiety T1  T2 | .082  .031 | **.278*****  **.313***** |
| Depression T1  T2 | .070  .023 | **.359*****  **.301***** |
| Flourishing T1  T2 | .052  .047 | **–.216****  **–.215**** |
| Inauthenticity T1  T2 | **.153***  –.087 | **.301*****  **.331***** |
| Relationship T1  Satisfaction T2 | –.120  –.105 | –.064  –.095 |
| Intimacy T1  T2 | –.079  –.128 | –.127  **–.162*** |

*Zero-Order Correlations Among Potential Moderators at T1 and Well-Being Outcomes*

*Note. *p* < .05, ***p* < .01, *** *p* < .001.

**Table 8S**

| Burden Predictor | *b* [95% CI] | *t* | *p* |
| --- | --- | --- | --- |
| *Daily Personal Impact* |  |  |  |
| **T1 flourishing** | **0.84 [0.76, 0.93]** | ***t*(154) = 19.59** | **<.001***** |
| burden score  **FNE**  burden*FNE | –0.33 [–0.71, 0.06]  **–0.55** **[–1.05, –0.05]**  0.11 [–0.02, 0.24] | *t*(154) = –1.68  ***t*(154) = –2.15**  *t*(154) = 1.70 | .095  **.033***  .091 |
| *Relationship Impact* |  |  |  |
| **T1 flourishing** | **0.84 [0.76, 0.93]** | ***t*(154) = 19.24** | **<.001***** |
| burden score  FNE  burden*FNE | –0.33 [–0.79, 0.14]  –0.65 [–1.35, 0.04]  0.11 [–0.04, 0.27] | *t*(154) = –1.39  *t*(154) = –1.85  *t*(154) = 1.49 | .167  .066  .138 |
| *Pressure to Reveal* |  |  |  |
| **T1 flourishing** | **0.84 [0.75, 0.92]** | ***t*(154) = 19.48** | **<.001***** |
| burden score  **FNE**  burden*FNE | –0.21 [–0.050, 0.08]  **–0.43** **[–0.77, 0.08]**  0.08 [–0.01, 0.17] | *t*(154) = –1.43  ***t*(154) =** –**2.44**  *t*(154) = 1.78 | .153  **.016***  .078 |
| *Anticipated Consequences* |  |  |  |
| **T1 flourishing** | **0.85 [0.76, 0.94]** | ***t*(154) = 19.20** | **<.001***** |
| burden score  FNE  burden*FNE | –0.10 [–0.50, 0.30]  –0.37 [–1.09, 0.35]  0.04 [–0.09, 0.18] | *t*(154) = –0.48  *t*(154) = –1.01  *t*(154) = 0.62 | .632  .313  .536 |
| *Overall Burden* |  |  |  |
| **T1 flourishing** | **0.84 [0.76, 0.93]** | ***t*(154) = 19.72** | **<.001***** |
| burden score  **FNE**  **burden*FNE** | –0.50 [–1.02, 0.02]  **–0.88 [–1.59, –0.16]**  **0.18 [0.01, 0.35]** | *t*(154) = –1.90  ***t*(154) = –2.43**  ***t*(154) = 2.07** | .059  **.016***  **.040*** |

*Predicting T2 Well-Being Outcomes from Burden Subscale & FNE Scores at T1*

*Note. *p* < .05, ***p* < .01, *** *p* < .001.

Analyses of simple slopes suggest that, for participants who were particularly high on fear of negative evaluation (i.e., +1 standard deviation), higher overall burden and higher burden on the Pressure to Reveal subscale predicted higher levels of flourishing, whereas there was no significant association between burden level and flourishing for those with average or below average scores on fear of negative evaluation (see Table 9S). That is, greater concern with social evaluation was associated with the opposite relationship between secrecy burden and flourishing that had been previously observed. Instead of adversely affecting well-being, more burdensome secrets seemed to entail some psychological benefits, perhaps because the secret-keeper was protecting themselves from what could otherwise be a highly distressing negative evaluation.

**Table 9S**

| Predictor | *b* [95% CI] | *t* | *p* |
| --- | --- | --- | --- |
| *Pressure to Reveal* |  |  |  |
| –1 SD FNE  Mean FNE  **+1 SD FNE** | –0.01 [–0.09, 0.07]  0.04 [–0.02, 0.10]  **0.09 [0.01, 0.17]** | *–*0.20  1.35  **2.32** | .84  .18  **.02*** |
| *Overall Burden* |  |  |  |
| –1 SD FNE  Mean FNE  **+1 SD FNE** | –0.06 [–0.08, 0.20]  0.04 [–0.06, 0.14]  **0.15 [0.01, 0.29]*** | *–*0.90  0.80  **1.96** | .37  .43  **.05*** |

*Summary of Simple Slope Analyses for Predicting T2 Flourishing from FNE & Burden Scores*

*Note. *p* < .05, ***p* < .01, *** *p* < .001. Simple slopes represent the slope between the burden score and T2 flourishing at each level of the predicted moderator, controlling for T1 flourishing.

**Reveal Status.** For our primary analyses, we excluded participants who revealed their secret to the target in some capacity between the first survey and two-week follow-up (*n*=56). However, in these exploratory analyses, we wanted to see if participants’ reveal status (i.e., whether or not they revealed the secret to the target or anyone else since the first survey) would moderate the observed effects of secrecy burden on relevant well-being outcomes. If keeping the secret is the primary source of burden, then revealing it should alleviate some of that burden and weaken the negative consequences for well-being.

First, we conducted 2x2 mixed measure ANOVAs for each well-being outcome, with assessment time (T1 or T2) as the within subjects variable and reveal status (0=did not reveal to anyone, 1=revealed secret in some capacity to someone) as the between subjects variable. However, we did not find any significant main effects of reveal status, nor any interactions between reveal status and assessment time. Next, we conducted a series of regression analyses in which we included the interaction between each burden subscale and participants’ reveal status as a predictor while still controlling for T1 levels of the outcome. For these analyses, we similarly coded participants who did not reveal the secret in any capacity to the target or anyone else as 0 (*n*=134) and any participants who revealed the secret in full to the target or other people, or partially to the target or other people, as 1 (*n*=56).

Reveal status did not interact with any of the secrecy burden subscales or overall burden when predicting anxiety and depression symptoms at T2. Similarly, reveal status did not interact with Daily Personal Impact or overall burden when predicting authenticity and flourishing at T2. However, as Table 10S shows, there were some significant and marginally significant interactions with the other subscales when predicting authenticity and flourishing. Analyses of simple slopes suggests that among participants who revealed their secret, higher burden from Anticipated Consequences was associated with higher feelings of inauthenticity (*b* = .40, *p* <.001), whereas for those who did not reveal the secret, Anticipated Consequences burden was not associated with feelings of authenticity (*b* = .08, *p* = .42). However, for the Relationship Impact burden subscale, we found the opposite relationships. Among participants who revealed their secret, higher burden from Relationship Impact was not associated with feelings of authenticity (*b* = 0.00, *p* = .98), whereas for participants who did not reveal the secret, higher burden from Relationship Impact was associated with higher feelings of inauthenticity (*b* = .32, *p* < .001). Lastly, for participants who revealed their secret, higher burden from Pressure to Reveal was associated with decreased flourishing (*b* = –.14, *p* = .01), whereas for those who did not reveal their secret, higher burden from Pressure to Reveal was not associated with flourishing (*b* = .05, *p =* .17).

**Table 10S**

| Outcome & Burden Predictors | *b* [95% CI] | *t* | *p* |
| --- | --- | --- | --- |
| *T2 Authenticity* |  |  |  |
| **Relationship impact**  **Reveal Status**  Rel. impact*Reveal Status | **0.32 [0.10, 0.54]**  **1.66 [0.01, 3.31]**  –0.32 [–0.68, 0.03] | ***t*(184) = 2.91**  ***t*(184) = 1.98**  *t*(184) = –1.78 | **.004****  **.049***  .076 |
|  |  |  |  |
| Anticipated consequences  Reveal Status  Anticip. conseq*Reveal Status | 0.08 [–0.12, 0.28]  –1.17 [–2.74, 0.40]  0.32 [–0.003, 0.64] | *t*(184) = 0.81  *t*(184) = –1.47  *t*(184) = 1.95 | .418  .143  .052 |
| *T2 Flourishing* |  |  |  |
| Pressure to Reveal  **Reveal Status**  **Pressure*Reveal Status** | 0.05 [–0.02, 0.12]  **0.74 [0.26, 1.21]**  –**0.18 [–0.31, –0.06]** | *t*(185) = 1.39  ***t*(185) = 3.05**  ***t*(185) = –2.98** | .168  **.003****  **.003**** |

*Predicting T2 Well-Being Outcomes from Burden Subscale & Reveal Status*

*Note. *p* < .05, ***p* < .01, *** *p* < .001. All analyses controlled for T1 levels of the well-being outcome (which were all significant). Each burden predictor and its interaction with reveal status was analyzed separately.

**Secret Characteristics.** Lastly, we wanted to see whether any characteristics of the secret itself (i.e., how serious or important it was, how long participants had been keeping it) moderated the effects of secrecy burden on well-being. Table 11S presents zero-order correlations among the secret characteristics, burden subscales, and our primary well-being outcomes.

**Table 11S**

*Zero-Order Correlations Among Secret Characteristics, Burden Subscales, and Well-Being*

|  | Length | Importance | Seriousness | Valence | Personal |
| --- | --- | --- | --- | --- | --- |
| *Burden Subscales* |  |  |  |  |  |
| Daily Personal  Impact | –.016 | **.290***** | **.396***** | **–.235**** | **.221**** |
| Relationship  Impact | –.023 | .012 | **.158*** | –.044 | .022 |
| Pressure to Reveal | **–.216**** | .032 | **.244**** | **–.245**** | .129 |
| Anticipated  Consequences | .036 | **.536***** | **.376***** | **–.425***** | **.383***** |
| Overall Burden | –.066 | **.351***** | **.464***** | **–.351***** | **.293***** |
| *Well-Being Outcomes* | |  |  |  |  |
| Anxiety T1  T2 | –.139  –.036 | **.223****  **.209**** | **.337*****  **.311***** | **–.257****  **–.255**** | .069  .125 |
| Depression T1  T2 | –.111  –.034 | **.171***  **.169*** | **.197***  **.235**** | **–.205****  **–.268***** | .054  .087 |
| Flourishing T1  T2 | –.053  –.030 | .018  –.031 | .003  –.013 | .072  .117 | .045  .070 |
| Inauthenticity T1  T2 | .058  .069 | .097  **.261***** | **.277*****  **.259**** | –.095  **–.228**** | .054  .095 |
| Relationship T1  Satisfaction T2 | –.055  –.065 | **.189***  **.163*** | .078  .032 | –.045  –.098 | .028  –.029 |
| Intimacy T1  T2 | –.054  –.001 | .119  .133 | .022  .051 | .030  –.092 | –.025  –.067 |

*Note. *p* < .05, ***p* < .01, *** *p* < .001.

Based on the zero-order correlations, none of the burden scores nor well-being outcomes at either time were associated with how long participants had been keeping the secret, with one exception that the longer participants had been keeping the secret, the lower they experienced Pressure to Reveal burden. Neither flourishing nor intimacy were associated with any of the secret characteristics, and relationship satisfaction was only associated with importance, such that the more important the secret was, the more satisfied participants were in their relationship with the target. None of the well-being outcomes were associated with how personal the secret was, although secrets that were more personal were associated with higher Daily Personal Impact, Anticipated Consequences, and overall burden.

More consistently, secrets that were higher in importance, seriousness, and negative information were associated with higher anxiety and depression symptoms at both timepoints and with lower authenticity at T2. These characteristics were also associated with higher Daily Personal Impact, Anticipated Consequences, and overall burden, and seriousness was additionally associated with higher Relationship Impact and Pressure to Reveal burden. The negativity of the secret was also associated with higher Pressure to Reveal burden.

Next, we conducted a series of regression analyses in which we included the interaction between each burden subscale and the secret characteristic (specifically length, importance, and seriousness) in predicting the well-being outcomes while controlling for T1 levels of the outcome. We chose length to rule out the possibility that the observed differences between T1 and T2 differed among people who had been keeping their secret for a longer or shorter period of time. As expected from the zero-order correlations, there were no significant main effects of length nor any interactions with any of the burden subscales. Thus, the observed outcomes were the same regardless of how long participants had been keeping their secret.

We next chose importance and seriousness because these characteristics were robustly associated with both the secrecy burden subscales and well-being outcomes. For anxiety and depression, neither importance nor seriousness had any main effects or significant interactions with any of the burden subscales. Thus, the effects of secrecy burden on anxiety and depression were consistent regardless of how serious or important the secret was. However, there was evidence of importance and seriousness having moderating effects on how Relationship Impact and Anticipated Consequences were associated with authenticity at T2 (see Table 12S).

**Table 12S**

| Outcome & Burden Predictors | *b* [95% CI] | *t* | *p* |
| --- | --- | --- | --- |
| *T2 Authenticity* |  |  |  |
| Relationship impact  Importance  **Rel. impact*Importance** | –0.33 [–0.82, 0.17]  –0.34 [–0.76, 0.08]  **0.11 [0.02, 0.20]** | *t*(153) = –1.29  *t*(153) = –1.58  ***t*(153) = 2.43** | .199  .117  **.016*** |
|  |  |  |  |
| Relationship impact  Seriousness  Rel. impact*Seriousness | –0.15 [–0.60, 0.31]  –0.30 [–0.74, 0.13]  0.09 [–0.18, 0.01] | *t*(153) = –0.64  *t*(153) = –1.36  *t*(153) = 1.77 | .524  .174  .078 |
| **Anticipated consequences**  **Seriousness**  Anticip. conseq.*Seriousness | **0.45 [0.10, 0.79]**  **0.41 [0.04, 0.79]**  –0.07 [–0.14, 0.001] | ***t*(153) = 2.54**  ***t*(153) = 2.16**  *t*(153) = –1.93 | **.012***  **.032***  .055 |

*Predicting T2 Inauthenticity from Burden Subscale & Secret Characteristics*

*Note. *p* < .05, ***p* < .01, *** *p* < .001. All analyses controlled for T1 levels of authenticity (which were all significant). Each burden predictor and its interaction with the secret characteristic was analyzed separately.

Analyses of simple slopes suggest that, for participants who had secrets that were particularly low in seriousness and importance (i.e., –1 standard deviation), higher burden on the Relationship Impact subscale had no association with T2 levels of inauthenticity. However, for secrets that were of average or above average seriousness and importance, higher Relationship Impact burden was associated with greater inauthenticity at T2 when controlling for T1 authenticity (see Table 13S). That is, people felt less authentic when their secret had a greater impact on their relationship with the target, but only for secrets that were of moderate or high importance and seriousness. Relational burden had no influence on well-being for trivial secrets.

Interestingly, seriousness had an additional and rather different moderating effect for Anticipated Consequences. Specifically, for participants with secrets that were particularly low in seriousness (i.e., –1 standard deviation), higher burden on Anticipated Consequences was associated with greater inauthenticity at T2. But, for participants with secrets of average or above average seriousness, Anticipated Consequences burden was not associated with T2 inauthenticity. That is, when participants expected negative consequences upon revealing, they did not feel less authentic if it was a serious secret but did feel less authentic for more trivial secrets. Indeed, it may be that people could rationalize their secrecy for serious matters such that it did not influence how authentic they perceived themselves to be with the target, but keeping a trivial secret with a high degree of potential consequences did feel inauthentic (i.e., it is more difficult to rationalize not disclosing trivial matters, especially if they would still somehow have big repercussions).

**Table 13S**

| Predictor | *b* [95% CI] | *t* | *p* |
| --- | --- | --- | --- |
| *Relationship Impact* |  |  |  |
| –1 SD importance  **Mean importance**  **+1 SD importance** | 0.04 [–0.21, 0.29]  **0.24 [0.06, 0.42]**  **0.45 [020, 0.70]** | 0.30  **2.61**  **3.57** | .76  **.01***  **.001***** |
| –1 SD seriousness  **Mean seriousness**  **+1 SD seriousness** | 0.07 [–0.18, 0.32]  **0.24 [0.04, 0.44]**  **0.40 [0.13, 0.67]** | 0.56  **2.44**  **2.90** | .58  **.02***  **.001***** |
| *Anticipated Consequences* |  |  |  |
| **–1 SD seriousness**  Mean seriousness  +1 SD seriousness | **0.26 [0.06, 0.46]**  0.12 [–0.06, 0.30]  *–*0.01 [–0.25, 0.23] | **2.54**  1.43  *–*0.12 | **.01***  .15  .91 |

*Simple Slope Analyses for Predicting T2 Inauthenticity from Secret Characteristics & Burden Scores*

*Note. *p* < .05, ***p* < .01, *** *p* < .001. Simple slopes represent the slope between the burden score and T2 inauthenticity at each level of the predicted moderator, controlling for T1 inauthenticity.

**References**

Brown, T.A. (2015). *Confirmatory factor analysis for applied research* (2^nd^ ed.). Guilford publications.

Cumming, G. (2014). The new statistics: Why and how. *Psychological science*, *25*(1), 7-29. <https://doi.org/10.1177/0956797613504966>s

Fleeson, W., & Wilt, J. (2010). The relevance of Big Five trait content in behavior to subjective authenticity: Do high levels of within‐person behavioral variability undermine or enable authenticity achievement? *Journal of Personality*, *78*(4), 1353-1382. <https://doi.org/10.1111/j.1467-6494.2010.00653.x>

Leary, M.R. (1983). A brief version of the Fear of Negative Evaluation Scale. *Personality and social psychology bulletin*, *9*(3), 371-375. <https://doi.org/10.1177/0146167283093007>

Slepian, M.L., & Moulton-Tetlock, E. (2019). Confiding secrets and well-being. *Social Psychological and Personality Science, 10*(4), 472-484. <https://doi.org/10.1177/1948550618765069>

Watson, D., & Clark, LA. (1994). *The PANAS-X: Manual for the positive and negative affect schedule-expanded form*. University of Iowa.
